# Efficacy and safety of interleukin inhibitors in the treatment of moderate-to-severe plaque psoriasis: a systematic review and network meta-analysis

**DOI:** 10.3389/fmed.2026.1766348

**Published:** 2026-07-23

**Authors:** June Ming, Yayi Jiang, Qinyao Wu, Xiaoyue Xiao, Mingling Chen

**Affiliations:** 1 Department of Dermatology, Sichuan Provincial Hospital of Traditional Chinese Medicine, Chengdu, Sichuan, China; 2 Department of Geriatrics, Sichuan Provincial Hospital of Traditional Chinese Medicine, Chengdu, Sichuan, China

**Keywords:** IL-17, IL23, interleukin inhibitors, meta-analysis, psoriasis

## Abstract

**Background:**

Psoriasis is a relapsing inflammatory skin disease that significantly impacts patients' quality of life, particularly those with moderate-to-severe lesions, which also impose considerable psychological stress. Biologics have become a primary therapeutic approach for moderate-to-severe plaque psoriasis. This study aims to evaluate the clinical efficacy and safety of biologics, providing guidance for clinical application.

**Methods:**

We searched PubMed, Cochrane Library, EMBASE, and Web of Science from inception to May 29, 2024, and conducted an updated supplementary search from May 30, 2024 to April 25, 2026 to identify randomized controlled trials (RCTs) assessing the efficacy and safety of IL-23/IL-17 inhibitors in treating moderate-to-severe plaque psoriasis. The quality of the included studies was evaluated using the Cochrane risk of bias tool, and a meta-analysis was performed using R software (version 4.3.1).

**Result:**

A total of 23 publications were included after screening, comprising 27 RCTs that were incorporated into the network meta-analysis. The meta-analysis results showed that several regimens, including Guselkumab 200 mg, Secukinumab 300 mg, and Brodalumab 210 mg, ranked highly for PASI 75 and PASI 90 at specific follow-up time points; however, ranking patterns varied across outcomes and follow-up durations. Additionally, Tildrakizumab 100 mg and Tildrakizumab 200 mg showed high SUCRA values for achieving cleared or almost cleared skin and reducing the Dermatology Life Quality Index (DLQI) score at most follow-up time points. In short-term safety analyses, some agents showed a higher incidence of adverse events compared to placebo, including Brodalumab 140 mg at 12 weeks [RR = 1.12, 95%CrI = (1.03, 1.23)] and Secukinumab 150 mg [RR = 1.22, 95% CrI = (1.09, 1.35)]. However, adverse-event reporting was not uniformly comprehensive across all included trials, and these findings should therefore be interpreted with caution.

**Conclusion:**

This network meta-analysis provides regimen-specific comparative evidence for short-term efficacy and safety of IL-23/IL-17 inhibitors. Treatment rankings should be interpreted within each outcome and follow-up time point, together with effect estimates, safety profiles, and the amount of supporting evidence.

**Systematic Review Registration:**

https://www.crd.york.ac.uk/PROSPERO, identifier CRD42024584441.

## 1 Introduction

As a chronic, immune-mediated inflammatory skin disorder, psoriasis is characterized by the appearance of red plaques and scaly lesions. It affects approximately 3.2% of adults and 0.13% of children in the United States, with an annual incidence of about 80 cases per 100,000 person-years. Globally, psoriasis affects approximately 125 million people, with prevalence varying by region, ranging from 0.5% in parts of Asia to as high as 8% in Norway. The disease is more common among Caucasians, with similar prevalence rates between men and women across most regions (1). Psoriasis is not only a superficial skin disorder but is also associated with numerous systemic comorbidities, including Crohn's disease, type 2 diabetes, metabolic syndrome, depression, and cardiovascular diseases (2). Among these, psoriatic arthritis (PsA) is the most notable, affecting up to 30% of psoriasis patients seen in dermatology clinics (3). PsA manifests as joint stiffness, pain, and swelling and can progress to debilitating joint destruction. Although psoriasis does not typically affect life expectancy, it substantially reduces quality of life. Recent studies indicate that at least one-third of psoriasis patients experience pathological anxiety, with interpersonal difficulties impacting daily life, leading to suicidal ideation in over 5% of cases (4).

Psoriasis is classified into four main clinical types: plaque, guttate, erythrodermic, and pustular. Plaque psoriasis is the most common type, accounting for 80%−90% of cases (1). It typically presents as well-demarcated red plaques with silvery-white scales, commonly affecting the scalp, elbows, knees, and lower back in a symmetrical distribution. Other types exhibit distinct features, such as guttate psoriasis with small droplet-like papules, erythrodermic psoriasis with widespread erythema and scaling, and pustular psoriasis characterized by sterile pustules.

The development and progression of psoriasis are influenced by genetic, environmental, and behavioral factors. Among these, genetic predisposition plays a pivotal role, while environmental and behavioral factors can exacerbate the condition. Immunological and genetic studies have identified interleukin-17 (IL-17), interleukin-23 (IL-23), and tumor necrosis factor-alpha (TNF-α) as central drivers of psoriasis pathogenesis. Biologic therapies targeting these cytokines have significantly improved treatment outcomes for moderate-to-severe plaque psoriasis and PsA (5). For mild psoriasis, treatment options include topical corticosteroids, vitamin D analogs, calcineurin inhibitors, keratolytics, and targeted phototherapy. For moderate-to-severe cases, the American Academy of Dermatology–National Psoriasis Foundation Guidelines recommend biologics as the primary treatment modality, combined with oral drugs, topical agents, or phototherapy as adjuncts.

Before the advent of biologics, oral medications, such as methotrexate, apremilast, acitretin, and cyclosporine, were widely used to treat moderate-to-severe plaque psoriasis. While effective for some patients, oral therapies generally offer lower efficacy than biologics, except for cyclosporine. Additionally, oral drugs have numerous contraindications and require close monitoring for adverse events (1). In contrast, topical therapies and phototherapy, although essential components of psoriasis treatment, are less convenient and effective than biologics.

In 2019, CY Erichsen et al. (6) published a systematic review evaluating the efficacy and safety of IL-23/IL-17-targeted biologics for moderate-to-severe plaque psoriasis. Their findings indicated that IL-17 inhibitors were more effective than IL-23 inhibitors but carried higher risks of adverse events. However, their meta-analysis primarily compared IL-23/IL-17 inhibitors with placebo and evaluated a single efficacy metrics. Therefore, we conducted an updated meta-analysis based on RCT studies using network meta-analysis to compare the drugs more comprehensively and assess the efficacy and safety of IL-23/IL-17 inhibitors for moderate-to-severe plaque psoriasis. Subgroup analyses were performed by different time points for outcome measures, aiming to provide new references for clinical decision-making.

## 2 Materials and methods

### 2.1 Search strategy

This study was independently conducted by two researchers who searched four databases: PubMed, Cochrane Library, EMBASE, and Web of Science. The search terms included “Psoriasis [MeSH Terms]” AND [“Interleukins (MeSH Terms)” OR “Brodalumab” OR “Ixekizumab” OR “Secukinumab” OR “Bimekizumab” OR “Guselkumab” OR “Risankizumab” OR “Tildrakizumab” OR “Ustekinumab [MeSH Terms]” OR “Netakimab” OR “Mirikizumab” OR “Izokibep” OR “HB0017”]. The search was conducted up to May 29, 2024, with no restrictions on region. Only studies published in English were included due to resource and feasibility considerations. The detailed search strategy can be found in Supplementary material 1. An updated literature search was conducted from May 30, 2024 to April 25, 2026 using the same search strategy and eligibility criteria (Supplementary material 2). Newly identified records were screened independently by two reviewers, and disagreements were resolved by discussion.

### 2.2 Inclusion and exclusion criteria

Studies meeting all of the following criteria were included: ➀ Study type: Randomized controlled trials (RCTs); ➂ Language: English literature; ➃Participants: Adult patients clinically diagnosed with moderate-to-severe plaque psoriasis who meet the indications for the use of interleukin inhibitors; ➄ Intervention: Treatment with interleukin inhibitors. Studies meeting any of the following criteria were excluded: ➀ Meta-analyses, reviews, systematic reviews, case reports, animal studies, studies with inappropriate controls, studies on non-psoriasis diseases, non-English literature, interventions not matching, retrospective studies, surgical interventions, letters, guidelines, conference abstracts; ➁ Studies on pathophysiology, observational studies, data lacking, non-randomized controlled trials, single-arm studies; ➂ Studies with unavailable full text.

### 2.3 Literature screening and data extraction

Two researchers strictly followed inclusion and exclusion criteria to retrieve the literature. They then used Endnote X9 software to manage all the references. After importing the retrieved articles into Endnote X9, duplicate publications were excluded. The researchers initiinally screened studies by title or abstract and then downloaded the full texts. After reading the full texts, they selected original studies that met the criteria for this systematic review. The basic information of the selected studies was extracted and cross-checked, with units of measurement standardized. In case of disagreements, the third researcher was consulted to reach a decision. The extracted information mainly included the title, first author, publication year, country, article type, sample size, patient age, intervention method, follow-up period, and outcome indicators.

### 2.4 Quality assessment

Two researchers independently assessed the literature and performed an initial screening based on inclusion and exclusion criteria. In cases of disagreement, a third researcher resolved disputes, and a final consensus was reached through discussion. The risk of bias of included randomized trials was assessed using the revised Cochrane risk-of-bias tool for randomized trials (RoB 2). The following domains were evaluated: bias arising from the randomization process, bias due to deviations from intended interventions, bias due to missing outcome data, bias in measurement of the outcome, and bias in selection of the reported result. Each study was judged as low risk of bias, some concerns, or high risk of bias.

### 2.5 Outcome measures

Primary outcomes: ➀ The proportion of participants achieving at least a 75% improvement in the Psoriasis Area and Severity Index from baseline (PASI 75); ➁ The proportion of participants achieving at least a 90% improvement in the Psoriasis Area and Severity Index from baseline (PASI 90); ➂ Change in Dermatology Life Quality Index (DLQI) score from baseline.

Secondary outcomes: ➀ The proportion of participants achieving cleared or almost cleared skin; ➁ Adverse events.

### 2.6 Statistical analysis

R software (version 4.3.1) was used to perform the network meta-analysis through the gemtc package (version 1.0–1) in conjunction with JAGS. A Bayesian framework based on Markov chain Monte Carlo (MCMC) simulation was applied. Four Markov chains were run with an initial value of 2.5, a thinning interval of 1, 5,000 burn-in iterations, and 20,000 iterations for model convergence. Model fit was assessed using the deviance information criterion (DIC). Global consistency and model adequacy were evaluated according to the available network structure. Each eligible dose regimen was defined as a separate intervention node because the aim of this network meta-analysis was to compare regimen-specific efficacy and safety rather than drug-class effects alone. Different doses of the same biologic agent were not merged, as they may represent distinct treatment strategies with different efficacy and safety profiles. Combining different doses into a single node could obscure dose-specific effects and introduce additional clinical heterogeneity. For binary outcomes, treatment effects were expressed as risk ratios (RRs) with 95% credible intervals (CrIs). For continuous outcomes, weighted mean differences (WMDs) with 95% CrIs were used. A treatment effect was considered statistically significant when the 95% CrI did not include 1 for binary outcomes or 0 for continuous outcomes. The analysis outputs included network plots, forest plots, ranking probability plots, SUCRA values, and league tables. SUCRA values were used to summarize the cumulative ranking probabilities of interventions. For efficacy outcomes, higher SUCRA values indicated a higher probability of better efficacy. For safety outcomes, SUCRA rankings were interpreted according to the direction of event risk. For binary outcomes, data were preferentially extracted from the intention-to-treat or modified intention-to-treat population. When ITT data were unavailable, the analysis population reported in the original trial was used. Missing outcome data were not additionally imputed by the review authors; instead, event counts and denominators were extracted as reported in the original trials. Binary outcomes were modeled using an arm-based binomial likelihood in the Bayesian network meta-analysis. Zero-event arms were retained in the primary network model without applying an arbitrary continuity correction. Publication bias and small-study effects were assessed visually using funnel plots where sufficient studies were available. Egger's test was not performed because most direct pairwise comparisons included fewer than 10 studies, which may lead to unreliable statistical inference. Local inconsistency was planned to be assessed using the node-splitting method when both direct and indirect evidence were available for the same comparison. However, node-splitting analysis was not feasible for comparisons without closed loops or without sufficient direct and indirect evidence. Therefore, inconsistency assessment was interpreted in the context of the available network structure. Because the primary analysis was conducted as a Bayesian network meta-analysis rather than a set of conventional pairwise meta-analyses, I^2^ values were not used as the primary heterogeneity measure. Moreover, many dose-specific direct comparisons were informed by only one or very few trials, making pairwise I^2^ not estimable or not informative. Therefore, model fit, consistency assessment, credible intervals, and the structure of available evidence were considered together when interpreting the robustness and reliability of the results.

### 2.7 Abbreviations for interventions

Drugs included in the analysis were assigned abbreviations as follows: Guselkumab (GUS), Tildrakizumab (TIL), Ustekinumab (UST), Brodalumab (BRO), Risankizumab (RIS), Secukinumab (SEC), Mirikizumab (MIR), Ixekizumab (IXE), Netakimab (NET), and Briakinumab (BRI).

## 3 Results

### 3.1 Literature search process

**Figure 1:**
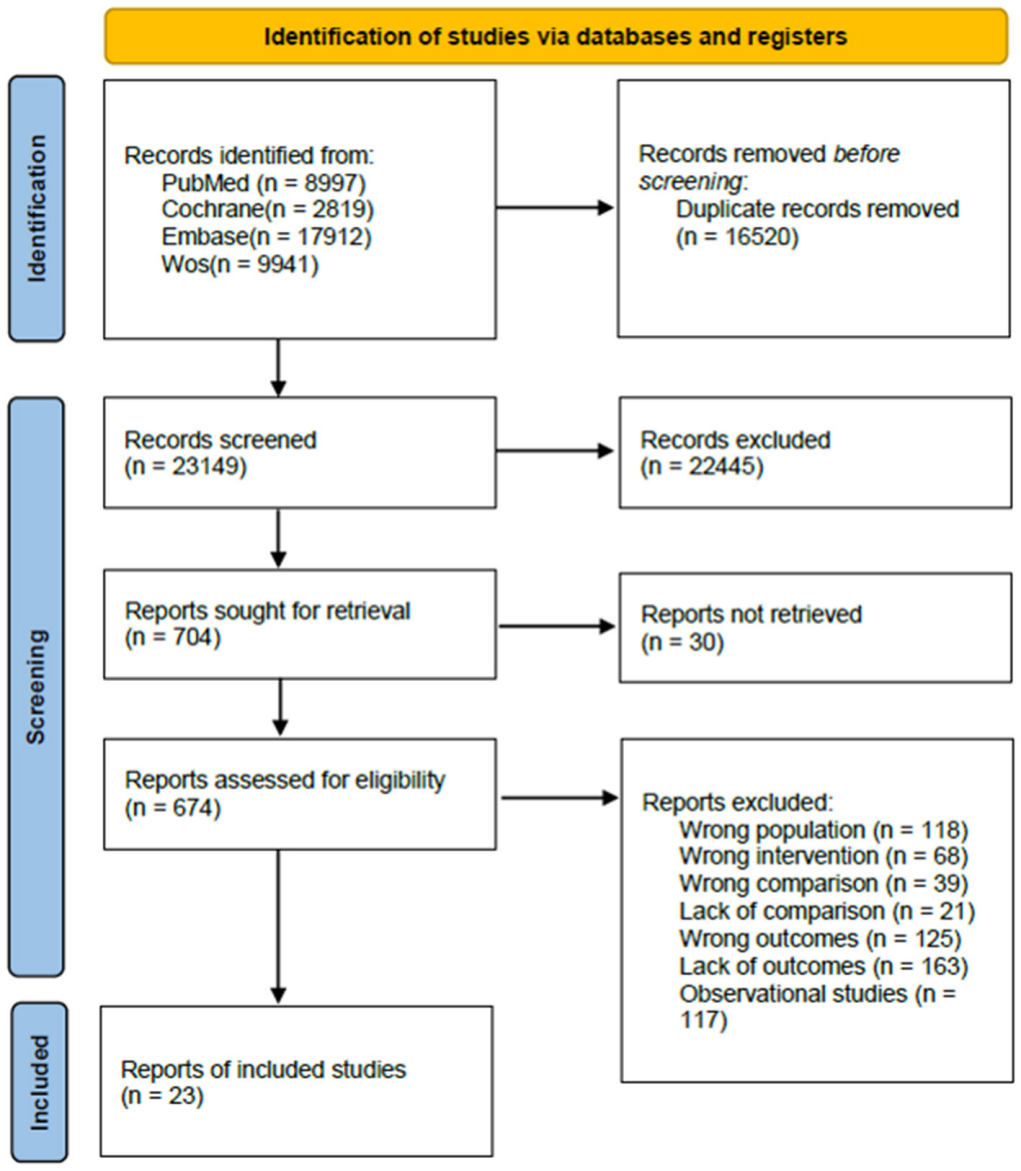
Literature selection flowchart.

A total of 39,669 studies were retrieved from the databases. After importing the articles into EndNote X9, 16,520 duplicate studies were removed. Titles and abstracts were screened, excluding 22,445 irrelevant studies. A further 681 articles were excluded after full-text review for not meeting inclusion criteria. Ultimately, 23 studies were included, comprising 27 RCTs that were eligible for the network meta-analysis. The literature selection process is illustrated in Figure 1. The updated search conducted from May 30, 2024 to April 25, 2026 identified 5,586 additional records. After screening, six potentially relevant studies were identified and summarized in Supplementary material 2. These studies were not incorporated into the primary quantitative synthesis because full incorporation would require reconstruction of the network and reanalysis of all outcomes. Therefore, the primary network meta-analysis remained based on 27 RCTs involving 13,935 participants.

### 3.2 Characteristics of included studies

A total of 23 publications reporting 27 RCTs (7–29) were included, involving 13,935 participants, with 10,254 in the experimental group and 3,681 in the placebo group. Some publications (e.g., phase III trials reported as A/B studies) presented results from two independent RCT cohorts, which were treated as separate trials in the network meta-analysis (7, 8, 12, 13, 27, 28, 30). All 27 RCTs included relevant intervention data and were published in English. The basic characteristics of the included studies are detailed in Table 1A. Clinically approved and commonly used dosing regimens are summarized separately in Table 1B Continued to facilitate rapid clinical interpretation of the comparative findings.

**Table 1A:** Basic characteristics of included studies.

References	Intervention	Country	Source of case	Trial registration number	Sample size		Age (mean ±SD)	Duration of follow-up	Outcome measures
					Gender (M/F)	Intervention grouping			
Blauvelt et al. (19)	Guselkumab vs. Placebo	Multi-country	Multi-center	NCT02207231	359/144	329 vs. 174	44.25 ± 12.79	16 week	➀➁➂➃➄➅
Ohtsuki et al. (20)	Guselkumab vs. Placebo	Japan	Multi-center	NCT02325219	145/47	128 vs. 64	48.75 ± 11.45	16 week	➀➁➂➃
Ferris et al. (10)	Guselkumab vs. Placebo	Multi-country	Multi-center	NCT02905331	53/25	62 vs. 16	46.04 ± 13.43	16 week	➀➁➃➄➅
Gordon et al. (11)	Guselkumab vs. Placebo	Multi-country	Multi-center	NCT01483599	177/73	208 vs. 42	–	16 week	➀➁➂➃
Papp et al. (21)	Tildrakizumab vs. Placebo	Multi-country	Multi-center	NCT01225731	270/85	309 vs. 46	44.93 ± 12.86	16 week	➀➁➂➄➅
Reich et al. (27)	Tildrakizumab vs. Placebo	Multi-country	Multi-center	NCT01722331	533/239	617 vs. 155	46.90 ± 13.21	12 week	➀➁➃➅➆➈➉
Reich et al. (28)	Tildrakizumab vs. Placebo	Multi-country	Multi-center	NCT01729754	557/220	621 vs. 156	44.96 ± 13.33	12 week	➀➁➃➄➅➆➈➉
Leonardi et al. (16)	Ustekinumab vs. Placebo	Multi-country	Multi-center	NCT00267969	531/235	511 vs. 255	45.27 ± 11.71	12 week	➀➁➂➃➄➅➆➇➈➉
Papp et al. (22)	Ustekinumab vs. Placebo	Multi-country	Multi-center	NCT00307437	840/390	820 vs. 410	46.23 ± 12.25	12 week	➀➁➂➃➄➅➆➇➈➉
Papp et al. (24)	Brodalumab vs. Placebo	Multi-country	Multi-center	NCT01708590	484/177	441 vs. 220	46.33 ± 12.66	12 week	➀➁➃➄➅➆➇➈➉
Lebwohl et al. (14)	Brodalumab vs. Placebo	Multi-country	Multi-center	NCT01708603	1053/478	1222 vs. 309	44.80 ± 12.99	12 week	➀➃➄➅➆
Lebwohl et al. (14)	Brodalumab vs. Placebo	Multi-country	Multi-center	NCT01708629	1076/492	1253 vs. 315	44.80 ± 12.99	12 week	➀➃➄➅➆
Odnopozova et al. (18)	Risankizumab vs. Placebo	Russia	Multi-center	NCT03518047	27/23	41 vs. 9	44.57 ± 12.86	16 week	➀➁➃➄➅
Nakagawa et al. (17)	Brodalumab vs. Placebo	Japan	Multi-center	NCT01748539	120/31	113 vs. 38	45.67 ± 11.88	12 week	➀➁➂➃➄➅➆➈➉
Langley et al. (13)	Secukinumab vs. Placebo	Multi-country	Multi-center	NCT01365455	509/229	490 vs. 248	45.07 ± 13.12	12 week	➀➁➃➄➆➇➈➉
Langley et al. (13)	Secukinumab vs. Placebo	Multi-country	Multi-center	NCT01358578	697/283	654 vs. 326	44.67 ± 12.90	12 week	➀➁➃➄➆➇➈➉
Paul et al. (25)	Secukinumab vs. Placebo	Multi-country	Multi-center	NCT01636687	125/57	121 vs. 61	44.72 ± 13.80	12 week	➀➁➃➄➆➇➈
Blauvelt et al. (9)	Secukinumab vs. Placebo	Multi-country	Multi-center	NCT01555125	117/60	118 vs. 59	45.87 ± 13.91	12 week	➀➁➃➄➅➆➇➈
Blauvelt et al. (7)	Mirikizumab vs. Placebo	Multi-country	Multi-center	NCT03482011	373/157	423 vs. 107	46.26 ± 13.61	16 week	➀➁➃
Gordon et al. (12)	Risankizumab vs. Placebo	Multi-country	Multi-center	NCT02684370	291/115	304 vs. 102	48.55 ± 13.44	16 week	➀➁➃
Gordon et al. (12)	Risankizumab vs. Placebo	Multi-country	Multi-center	NCT02684357	270/122	294 vs. 98	46.23 ± 13.58	16 week	➀➁➃
Papp et al. (23)	Brodalumab vs. Placebo	Multi-country	Multi-center	NCT00975637	127/71	160 vs. 38	42.45 ± 12.23	12 week	➀➁➂➃➄➅➆➇➉
Leonardi et al. (15)	Ixekizumab vs. Placebo	Multi-country	Multi-center	NCT01107457	81/61	115 vs. 27	46.21 ± 12.94	16 week	➀➁➃
Puig et al. (26)	Netakimab vs. Placebo	Russian,Belarus	Multi-center	NCT03390101	156/57	169 vs. 44	41.18 ± 13.54	12 week	➀➁
Reich et al. (27)	Guselkumab vs. Placebo	Multi-country	Multi-center	NCT02207244	522/222	496 vs. 248	43.57 ± 12.26	16 week	➀➁➂➄➅
Strober et al. (29)	Briakinumab vs. Placebo	America	Multi-center	NCT00710580	139/72	139 vs. 72	44.93 ± 13.22	8 week	➀➁➃
Blauvelt et al. (36)	Risankizumab vs. Placebo	America	Multi-center	NCT03875482	87/70	105 vs. 52	47.88 ± 15.32	16 week	➁

Outcome measures: PASI 75; PASI 90; DLQI; achieving cleared or almost cleared skin; AE; SAE; AE leading to discontinuation; Headache; Nasopharyngitis; Upper respiratory tract infection. PASI, Psoriasis Area and Severity Index; AE, adverse event; SAE, serious adverse event; RCT, randomized controlled trial; PBO, placebo.

**Table 1B:** Clinically approved and commonly used dosing regimens of included interleukin inhibitors for moderate-to-severe plaque psoriasis.

Drug	Target	Induction regimen	Maintenance regimen	Approval status
Secukinumab (SEC)	IL-17A	300 mg weekly at Weeks 0–4	300 mg every 4 weeks	Approved
Ixekizumab (IXE)	IL-17A	160 mg at Week 0; 80 mg every 2 weeks through Week 12	80 mg every 4 weeks	Approved
Brodalumab (BRO)	IL-17RA	210 mg at Weeks 0,1,2	210 mg every 2 weeks	Approved
Guselkumab (GUS)	IL-23 p19	100 mg at Weeks 0,4	100 mg every 8 weeks	Approved
Risankizumab (RIS)	IL-23 p19	150 mg at Weeks 0,4	150 mg every 12 weeks	Approved
Tildrakizumab (TIL)	IL-23 p19	100 mg at Weeks 0,4	100 mg every 12 weeks	Approved
Ustekinumab (UST)	IL-12/23 p40	45 mg ( ≤ 100 kg) or 90 mg (>100 kg) at Weeks 0,4	Same dose every 12 weeks	Approved

Higher or alternative dosing regimens included in certain trials (e.g., SEC 150 mg, GUS 200 mg, TIL 200 mg) represent investigational or non-standard doses and are analyzed separately within the network but are not current guideline-recommended maintenance regimens.

### 3.3 Quality assessment

**Figure 2:**
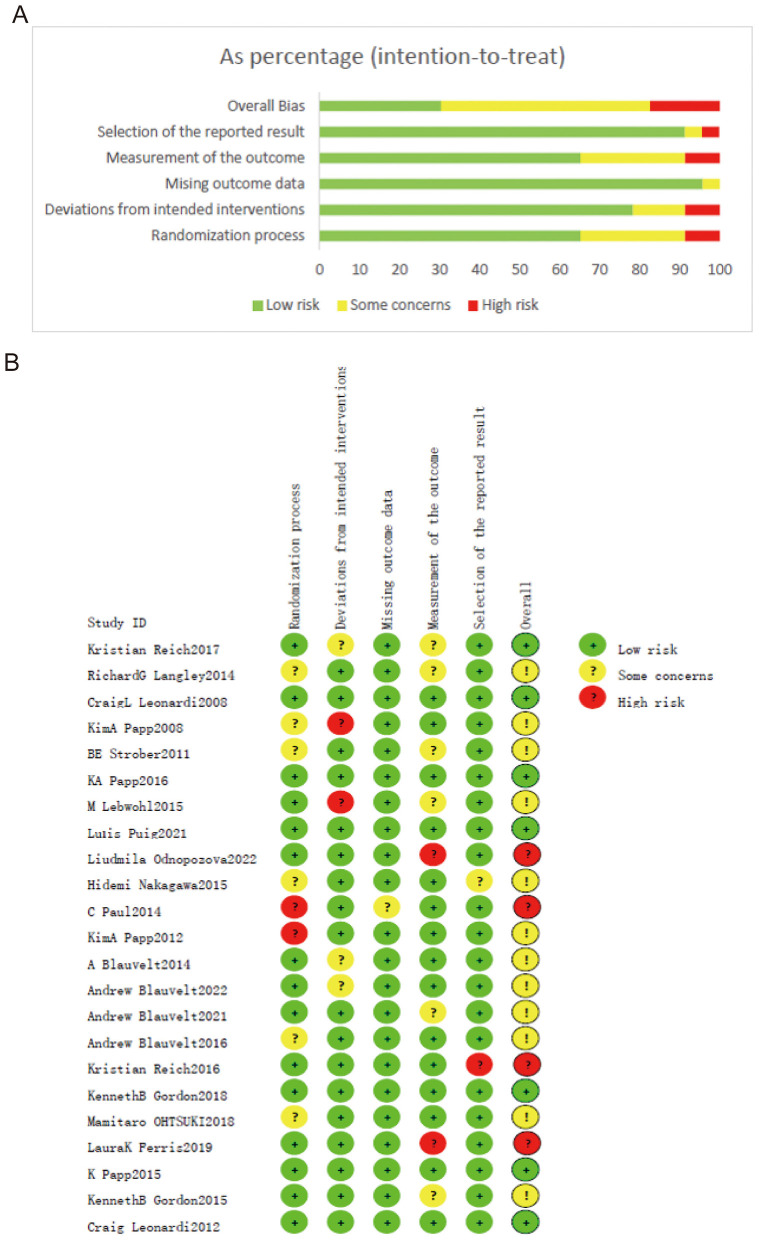
Risk of bias assessment diagram. **(A)** Risk of bias summary; **(B)** Risk of bias graph.

Among the included studies, two (23, 25) did not describe the randomization process and were assessed as having a high risk of bias. Two studies (14, 22) had a high risk of bias in deviation from intended interventions, and another two studies (10, 18) had bias in outcome measurements. One study (27) was identified as having high reporting bias due to incomplete results. Despite these issues, most studies were rated as low risk across the various dimensions, and the overall quality of the included literature was deemed high (Figure 2).

### 3.4 Network meta-analysis of included studies

In addition to ranking metrics (SUCRA), the magnitude of treatment effects vs. placebo was considered to enhance clinical interpretability. Across most follow-up time points, several IL-23 and IL-17 inhibitors demonstrated substantial relative improvements compared with placebo, suggesting clinically meaningful increases in response probability.

#### 3.4.1 Meta-analysis results for PASI 75

**Figure 3:**
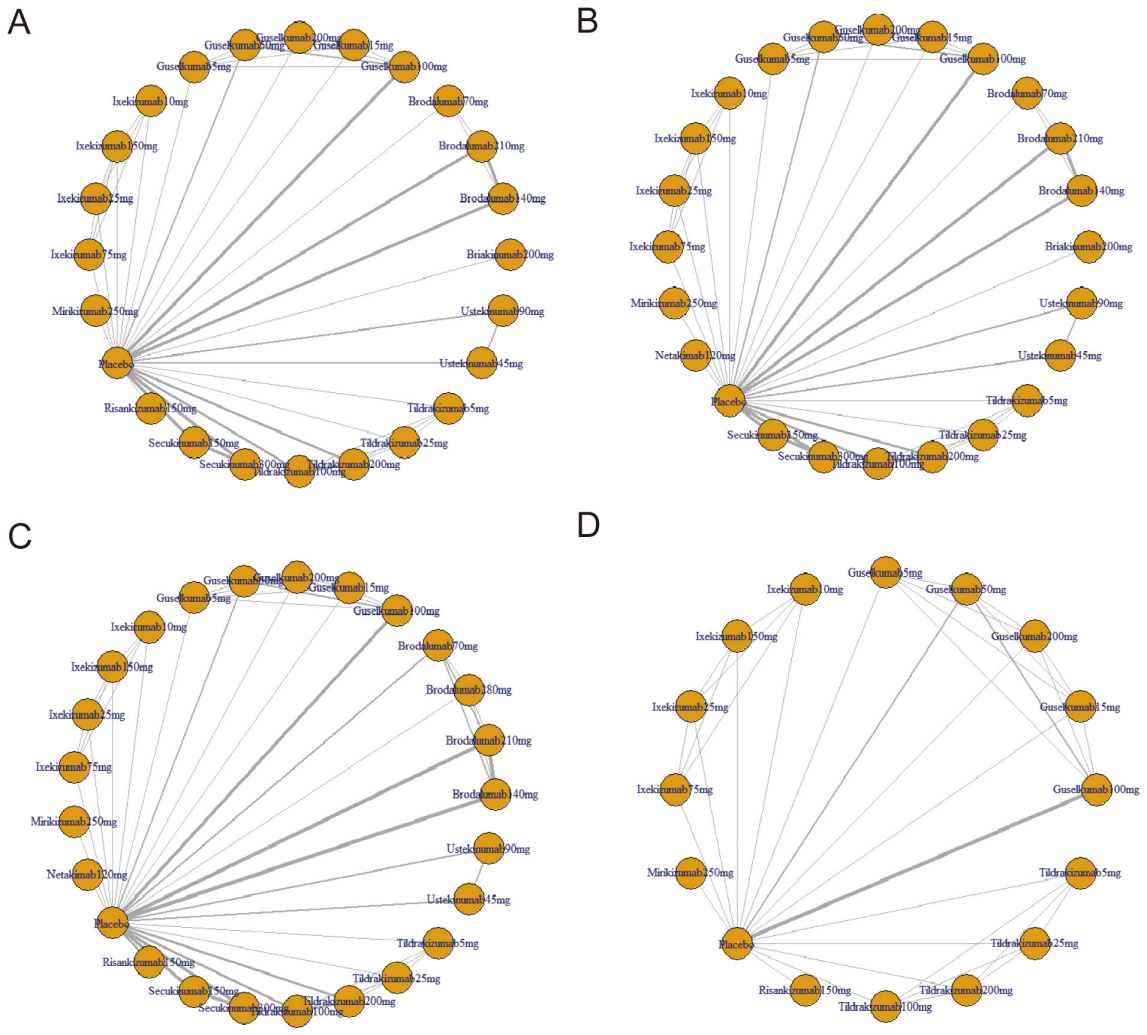
PASI75 meta-network diagram. **(A)** The PASI75 network was followed up for 4 weeks. **(B)** The PASI75 network was followed up for 8 weeks. **(C)** The PASI75 network was followed up for 12 weeks. **(D)** The PASI75 network was followed up for 16 weeks.

A network meta-analysis 22 (7–21, 23–29) studies including 26 RCTs was conducted for the PASI 75 outcome, involving 26 interventions and 13,778 participants, including 10,149 in the treatment groups and 3,629 in the placebo groups (Figure 3). Additionally, subgroup analyses based on follow-up duration were performed to explore how the intervention effects changed over time.

At 4 weeks of follow-up (Figure 3A), the league table showed no statistically significant differences between GUS-200 mg, IXE-10 mg, RIS-150 mg, TIL-5 mg, and placebo, while other IL-23/IL-17 inhibitors demonstrated significantly more participants achieving PASI 75 compared to placebo. Among all interventions, the highest SUCRA rankings were SEC-300 mg (84.10%), UST-90 mg (83.99%), and BRO-210 mg (82.60%). Comparisons of SUCRA-ranked top interventions revealed that BRO-140 mg [RR = 3.86, 95%CrI = (1.89, 10.36)] and BRO-210mg [RR = 5.04, 95%CrI = (2.48, 13.57)] significantly increased the number of participants achieving PASI 75 compared to BRO-70 mg, while SEC-150 mg significantly reduced PASI 75 attainment compared to SEC-300 mg [RR = 0.59, 95%CrI = (0.48, 0.72)]. In some within-agent comparisons, dose-specific ranking patterns were observed; for example, BRO-210 mg ranked above BRO-140 mg and BRO-70 mg, and SEC-300 mg ranked above SEC-150 mg. These findings were descriptive and were not based on formal dose-response testing. Other comparisons showed GUS-100 mg (46.61%) ranked higher than GUS-5 mg, 15 mg, 50 mg, and 200 mg; IXE-150 mg (77.24%) ranked higher than IXE-10 mg, 25 mg, and 75 mg; and TIL-25 mg (55.32%) ranked higher than TIL-5 mg, 100 mg, and 200 mg (Tables 2, 3).

**Table 2:**
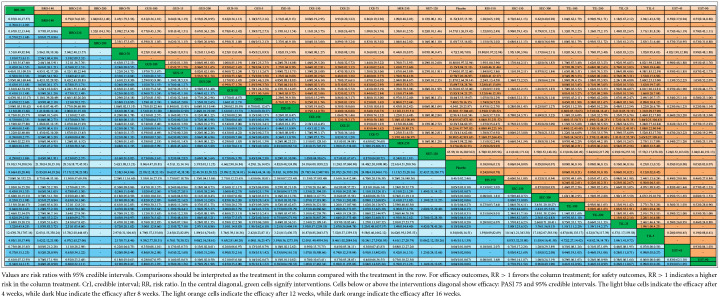
PASI75 network meta-analysis result.

**Table 3:** Results of SUCRA of PASI 75.

Intervention	SUCRA (%)			
	4 weeks	8 weeks	12 weeks	16 weeks
BRI-200	67.21	31.92	–	–
BRO-140	72.14	41.91	41.21	–
BRO-210	82.6	55.16	59.09	–
BRO-280	–	–	38.09	–
BRO-70	30.81	13	9.95	–
GUS-100	46.61	70.57	74.63	53.33
GUS-15	25.76	51.03	70.37	51.33
GUS-200	19.49	68.63	85.72	57.26
GUS-50	37.12	73.04	74.01	58.58
GUS-5	24.96	43.82	36.31	24.55
IXE-10	35.58	34.94	16.34	41.44
IXE-150	77.24	74.51	53.97	77.39
IXE-25	55.09	58.88	49.84	71.56
IXE-75	62.05	66.7	54.88	80.33
MIR-250	72	35.8	39.72	35.58
NET-120	–	75.41	92.46	–
Placebo	3.05	0.37	0.03	0.16
RIS-150	21.98	–	31.96	18.87
SEC-150	67.21	67.52	64.32	–
SEC-300	84.1	80.23	75.9	–
TIL-100	46.1	36.38	39.07	63.45
TIL-200	40.04	38.67	44.58	71.38
TIL-25	55.32	19.81	30.56	61.59
TIL-5	13.02	5.42	8.26	33.2
UST-45	76.52	75.23	76.03	–
UST-90	83.99	81.05	82.68	–

At 8 weeks of follow-up (Figure 3B), the league table showed that TIL-5 mg had no statistically significant difference compared to placebo, while other IL-23/IL-17 inhibitors showed significantly more participants achieving PASI 75 compared to placebo. The highest SUCRA rankings were UST-90 mg (81.05%) and SEC-300 mg (80.23%). Comparisons of SUCRA-ranked top interventions indicated that SEC-150 mg significantly reduced PASI 75 attainment compared to SEC-300 mg [RR = 0.80, 95%CrI = (0.73, 0.88)], and SUCRA values confirmed that SEC-300 mg ranked higher than SEC-150 mg. Other results showed BRO-210 mg (55.16%) ranked higher than BRO-70 mg and 140 mg; GUS-50 mg (73.04%) ranked higher than GUS-5 mg, 15 mg, 100 mg, and 200 mg; IXE-150 mg (74.51%) ranked higher than IXE-10 mg, 25 mg, and 75 mg; and TIL-200 mg (38.67%) ranked higher than TIL-5 mg, 25 mg, and 100 mg (Tables 2, 3).

At 12 weeks of follow-up (Figure 3C), all IL-23/IL-17 inhibitors significantly increased the number of participants achieving PASI 75 compared to placebo. The magnitude of these effects was substantial across most active treatments, indicating clinically meaningful improvements in lesion reduction beyond statistical significance. The highest SUCRA rankings were NET-120 mg (92.46%), GUS-200 mg (85.72%), and UST-90 mg (82.68%). Comparisons of SUCRA-ranked top interventions showed that GUS-100 mg [RR = 1.80, 95%CrI = (1.25, 2.75)], GUS-15 mg [RR = 1.72, 95%CrI = (1.15, 2.67)], GUS-200mg [RR = 2.09, 95%CrI = (1.46, 3.19)], and GUS-50 mg [RR = 1.79, 95%CrI = (1.24, 2.78)] significantly increased PASI 75 attainment compared to GUS-5 mg, and SUCRA values confirmed that GUS-5 mg ranked lower than GUS-15 mg, 50 mg, 100 mg, and 200 mg. Other comparisons showed BRO-210 mg (59.09%) ranked higher than BRO-70 mg, 140 mg, and 280 mg; IXE-75 mg (54.88%) ranked higher than IXE-10 mg, 25 mg, and 150 mg; SEC-300 mg (75.90%) ranked higher than SEC-150 mg; and TIL-200 mg (44.58%) ranked higher than TIL-5 mg, 25 mg, and 100 mg (Tables 2, 3).

At 16 weeks of follow-up (Figure 3D), the league table showed that all IL-23/IL-17 inhibitors significantly increased PASI 75 attainment compared to placebo. The highest SUCRA rankings were IXE-75 mg (80.33%), IXE-150 mg (77.39%), and IXE-25 mg (71.56%). Comparisons of SUCRA-ranked top interventions revealed that IXE-10 mg significantly reduced PASI 75 attainment compared to IXE-150 mg [RR = 0.35, 95%CrI = (0.14, 0.78)], IXE-25 mg [RR = 0.40, 95%CrI = (0.16, 0.88)], and IXE-75 mg [RR = 0.34, 95%CrI = (0.13, 0.73)], and SUCRA values confirmed that IXE-10 mg ranked lower than IXE-25 mg, 75 mg, and 150 mg. Other results showed GUS-50 mg (58.58%) ranked higher than GUS-5 mg, 15 mg, 100 mg, and 200 mg; and TIL-200 mg (71.38%) ranked higher than TIL-5 mg, 25 mg, and 100 mg (Tables 2, 3).

#### 3.4.2 Meta-analysis results of PASI 90

**Figure 4:**
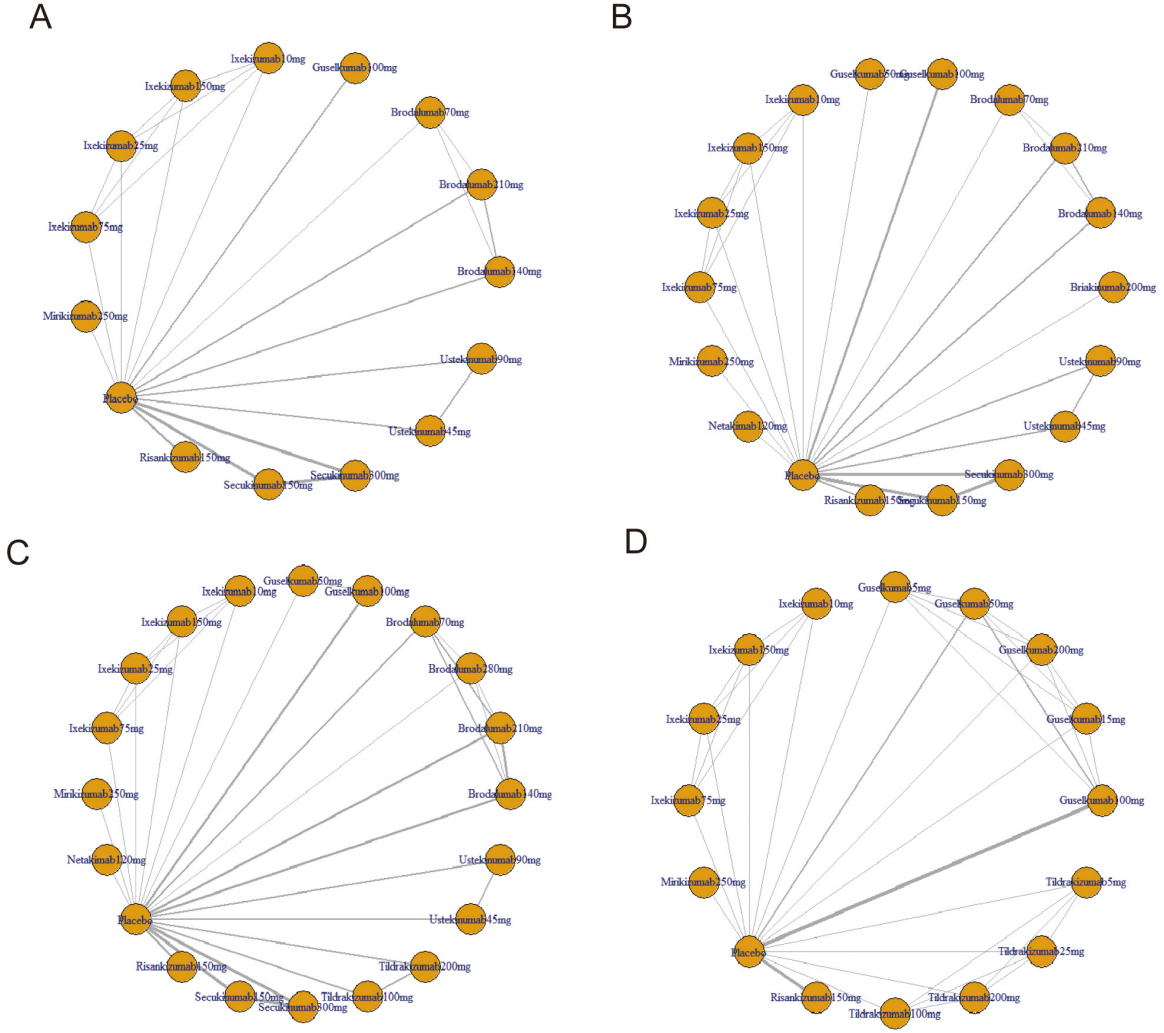
PASI90 meta-network diagram. **(A)** The PASI90 network was followed up for 4 weeks. **(B)** The PASI90 network was followed up for 8 weeks. **(C)** The PASI90 network was followed up for 12 weeks. **(D)** The PASI90 network was followed up for 16 weeks.

A network meta-analysis was conducted for the PASI 90 outcome involving 22 studies (7–21, 23–29), 25 randomized controlled trials, 26 interventions, and 10,832 patients, including 7,779 in the treatment groups and 3,053 in the placebo groups (Figure 4). Additionally, subgroup analyses were performed based on different follow-up durations to explore how the intervention effects varied over time.

At 4 weeks of follow-up (Figure 4A), the league table results showed no statistically significant differences between BRO-70 mg, GUS-100 mg, IXE-10 mg, IXE-25 mg, IXE-75 mg, MIR-250 mg, and placebo. However, other IL-23/IL-17 inhibitors showed significant differences in the number of patients achieving PASI 90 compared to placebo. Among all interventions, the highest SUCRA rankings were BRO-210 mg (93.36%), BRO-140 mg (83.35%), and SEC-300 mg (77.58%). Based on the league table, SEC-150 mg significantly reduced the number of patients achieving PASI 90 compared to SEC-300 mg [RR = 0.41, 95%CrI = (0.24, 0.65)], and SUCRA confirmed that SEC-300 mg ranked higher than SEC-150 mg. Additionally, the SUCRA value for IXE-150 mg (64.61%) was higher than for IXE (10 mg, 25 mg, 75 mg), and UST-90 mg (70.39%) ranked higher than UST-45 mg (Tables 4, 5).

**Table 4:**
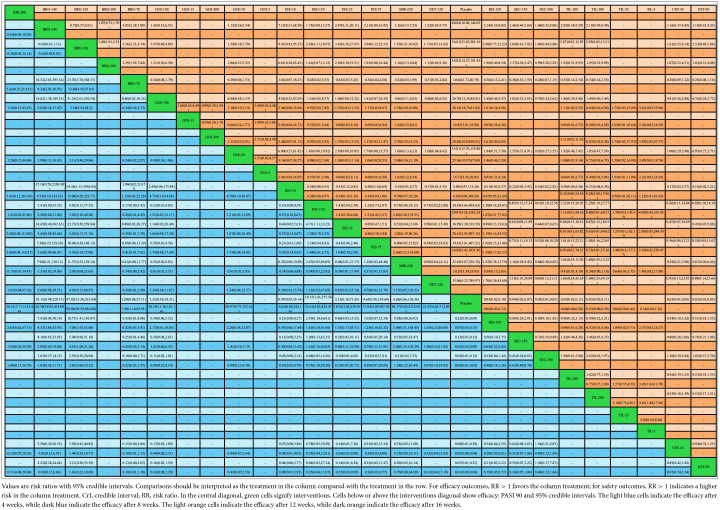
PASI90 network meta-analysis results.

**Table 5:** Results of SUCRA of PASI 90.

Intervention	SUCRA (%)			
	4 weeks	8 weeks	12 weeks	16 weeks
BRI-200	67.21	31.92	–	–
BRO-140	72.14	41.91	41.21	–
BRO-210	82.6	55.16	59.09	–
BRO-280	–	–	38.09	–
BRO-70	30.81	13	9.95	–
GUS-100	46.61	70.57	74.63	53.33
GUS-15	25.76	51.03	70.37	51.33
GUS-200	19.49	68.63	85.72	57.26
GUS-50	37.12	73.04	74.01	58.58
GUS-5	24.96	43.82	36.31	24.55
IXE-10	35.58	34.94	16.34	41.44
IXE-150	77.24	74.51	53.97	77.39
IXE-25	55.09	58.88	49.84	71.56
IXE-75	62.05	66.7	54.88	80.33
MIR-250	72	35.8	39.72	35.58
NET-120	–	75.41	92.46	–
Placebo	3.05	0.37	0.03	0.16
RIS-150	21.98	–	31.96	18.87
SEC-150	67.21	67.52	64.32	–
SEC-300	84.1	80.23	75.9	–
TIL-100	46.1	36.38	39.07	63.45
TIL-200	40.04	38.67	44.58	71.38
TIL-25	55.32	19.81	30.56	61.59
TIL-5	13.02	5.42	8.26	33.2
UST-45	76.52	75.23	76.03	–

At 8 weeks of follow-up (Figure 4B), the league table showed no statistically significant differences between IXE-10 mg and placebo. However, other IL-23/IL-17 inhibitors demonstrated significant improvements in PASI 90 attainment compared to placebo. The highest SUCRA rankings were BRO-210 mg (85.93%), UST-90 mg (78.66%), and UST-45 mg (76.89%). The league table revealed that BRO-140 mg [RR = 8.34, 95%CrI = (2.91, 36.93)] and BRO-210 mg [RR = 13.06, 95%CrI = (4.58, 57.85)] significantly increased PASI 90 attainment compared to BRO-70 mg, with BRO-210 mg ranking higher than BRO (70 mg, 140 mg). Moreover, GUS-50 mg (53.63%) ranked higher than GUS-100 mg, IXE-150 mg (55.18%) ranked higher than IXE (10 mg, 25 mg, 75 mg), and SEC-300 mg (57.97%) ranked higher than SEC-150 mg (Tables 4, 5).

At 12 weeks of follow-up (Figure 4C), with the exception of IXE-10 mg, most IL-23/IL-17 inhibitors demonstrated significant and pronounced improvements in PASI 90 attainment compared with placebo. The observed relative effects suggest a considerable increase in the likelihood of achieving near-complete skin clearance in clinical settings. The highest SUCRA rankings were BRO-210 mg (84.93%), SEC-300 mg (70.35%), and BRO-140 mg (68.82%). The league table indicated that BRO-210 mg significantly increased PASI 90 attainment compared to BRO-280 mg [RR = 1.49, 95%CrI = (1.01, 2.33)], whereas BRO-140 mg significantly reduced PASI 90 attainment compared to BRO-210 mg [RR = 0.72, 95%CrI = (0.57, 0.91)], and SEC-150 mg significantly reduced PASI 90 attainment compared to SEC-300 mg [RR = 0.72, 95%CrI = (0.59, 0.89)]. SUCRA values confirmed that BRO-210 mg ranked higher than BRO (140 mg, 280 mg), and SEC-300 mg ranked higher than SEC-150 mg. Additionally, GUS-50 mg (65.23%) ranked higher than GUS-100 mg, IXE-150 mg (54.43%) ranked higher than IXE (10 mg, 25 mg, 75 mg), TIL-100 mg (38.10%) ranked higher than TIL-200 mg, and UST-90 mg (68.09%) ranked higher than UST-45 mg (Tables 4, 5).

At 16 weeks of follow-up (Figure 4D), the league table showed no statistically significant differences between IXE-10 mg, TIL-5 mg, and placebo, while other IL-23/IL-17 inhibitors showed significant differences compared to placebo. The highest SUCRA rankings were TIL-200 mg (72.80%), GUS-100 mg (70.72%), and GUS-200 mg (69.77%). Additionally, IXE-150 mg (68.75%) ranked higher than IXE (10 mg, 25 mg, 75 mg) (Tables 4, 5).

#### 3.4.3 Achieving cleared or almost cleared skin

**Figure 5:**
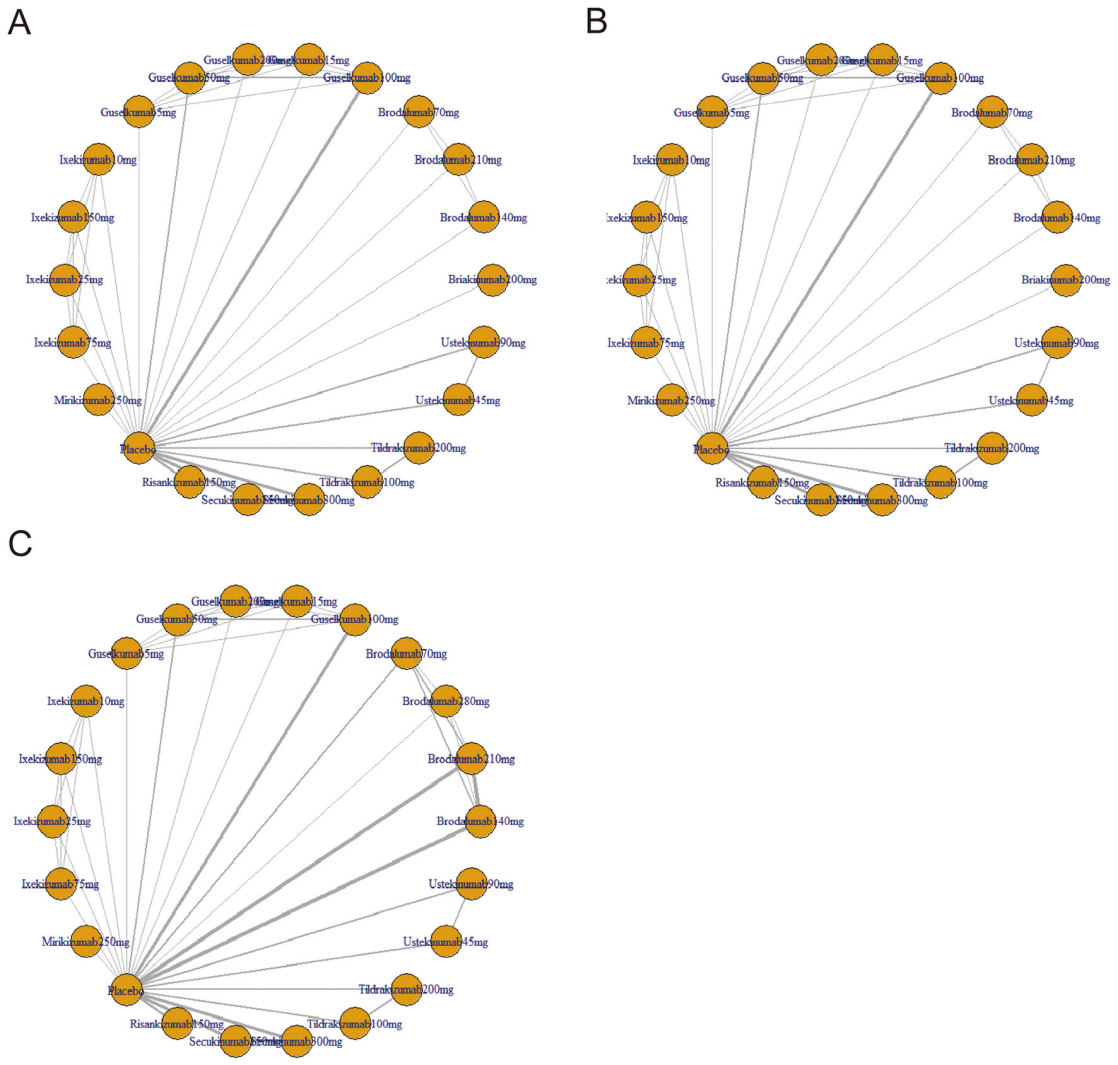
Achieving cleared or almost cleared skin meta-network diagram. **(A)** The achieving cleared or almost cleared skin network was followed up for 4 weeks. **(B)** The achieving cleared or almost cleared skin network was followed up for 8 weeks. **(C)** The achieving cleared or almost cleared skin network was followed up for 12 weeks.

For skin clearance outcomes, a network meta-analysis was conducted involving 19 studies (11, 16, 17, 19–23, 27) 23 interventions across 23 RCTs with a total of 12,471 patients, including 9,178 in the treatment groups and 3,293 in the placebo groups (Figure 5). Subgroup analyses were performed based on different follow-up times.

At 8 weeks of follow-up (Figure 5B), IL-23/IL-17 inhibitors demonstrated a statistically significant increase in the number of patients achieving cleared or almost cleared skin compared to placebo. The highest SUCRA rankings were SEC-300 mg (94.70%), SEC-150 mg (83.75%), and GUS-50 mg (78.76%). Comparing different doses of highly ranked interventions, GUS-15 mg [RR = 0.49, 95%CrI = (0.27, 0.80)] and GUS-50 mg [RR = 0.49, 95%CrI = (0.27, 0.79)] significantly reduced the number of patients achieving skin clearance compared to GUS-200 mg, and SEC-150 mg significantly reduced skin clearance compared to SEC-300 mg [RR = 0.69, 95%CrI = (0.59, 0.79)]. SUCRA rankings indicated that GUS-15 mg ranked lower than GUS (50 mg, 200 mg), and SEC-300 mg ranked higher than SEC-150 mg. For lower-ranked doses, BRO-210 mg (63.07%) ranked higher than BRO (70 mg, 140 mg), IXE-150 mg (44.64%) ranked higher than IXE (10 mg, 25 mg, 75 mg), TIL-100 mg (28.84%) ranked higher than TIL-200 mg, and UST-90 mg (51.03%) ranked higher than UST-45 mg (Tables 6, 7).

**Table 6:**
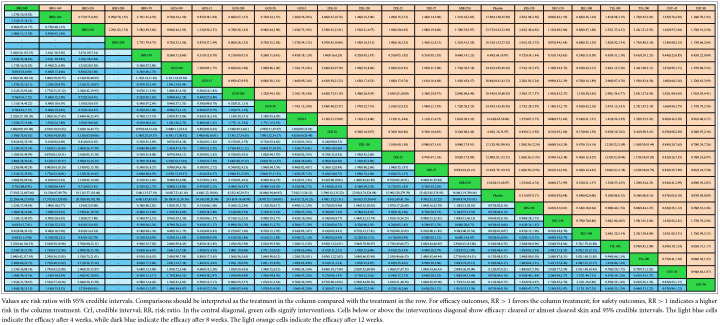
Cleared or almost cleared skin network meta-analysis results.

**Table 7:** Results of SUCRA of cleared or almost cleared skin.

Intervention	SUCRA (%)			
	4 weeks	8 weeks	12 weeks	16 weeks
BRI-200	67.21	31.92	–	–
BRO-140	72.14	41.91	41.21	–
BRO-210	82.6	55.16	59.09	–
BRO-280	–	–	38.09	–
BRO-70	30.81	13	9.95	–
GUS-100	46.61	70.57	74.63	53.33
GUS-15	25.76	51.03	70.37	51.33
GUS-200	19.49	68.63	85.72	57.26
GUS-50	37.12	73.04	74.01	58.58
GUS-5	24.96	43.82	36.31	24.55
IXE-10	35.58	34.94	16.34	41.44
IXE-150	77.24	74.51	53.97	77.39
IXE-25	55.09	58.88	49.84	71.56
IXE-75	62.05	66.7	54.88	80.33
MIR-250	72	35.8	39.72	35.58
NET-120	–	75.41	92.46	–
Placebo	3.05	0.37	0.03	0.16
RIS-150	21.98	–	31.96	18.87
SEC-150	67.21	67.52	64.32	–
SEC-300	84.1	80.23	75.9	–
TIL-100	46.1	36.38	39.07	63.45
TIL-200	40.04	38.67	44.58	71.38
TIL-25	55.32	19.81	30.56	61.59
TIL-5	13.02	5.42	8.26	33.2
UST-45	76.52	75.23	76.03	–
UST-90	83.99	81.05	82.68	–

At 12 weeks of follow-up (Figure 5C), IL-23/IL-17 inhibitors continued to show significant improvements in skin clearance compared to placebo, with large relative treatment effects indicating clinically meaningful gains in achieving clear or almost clear skin. The highest SUCRA rankings were GUS-200 mg (91.66%), SEC-300 mg (87.08%), and BRO-210 mg (81.14%). Comparing different doses of highly ranked interventions, BRO-210 mg significantly increased skin clearance compared to BRO-280 mg [RR = 1.29, 95%CrI = (1.02, 1.74)], while BRO-140 mg [RR = 0.77, 95%CrI = (0.71, 0.85)] and SEC-150 mg [RR = 0.79, 95%CrI = (0.70, 0.88)] significantly reduced skin clearance compared to BRO-210 mg and SEC-300 mg, respectively. SUCRA rankings confirmed that BRO-210 mg ranked higher than BRO (140 mg, 280 mg), and SEC-300 mg ranked higher than SEC-150 mg. For lower-ranked doses, IXE-75 mg (38.80%) ranked higher than IXE (10 mg, 25 mg, 150 mg), TIL-200 mg (32.14%) ranked higher than TIL-100 mg, and UST-90 mg (53.71%) ranked higher than UST-45 mg (Tables 6, 7).

At 4 weeks of follow-up (Figure 5A), the league table showed no statistically significant differences between IXE-10 mg and placebo. However, other IL-23/IL-17 inhibitors demonstrated a statistically significant increase in the number of patients achieving cleared or almost cleared skin compared to placebo. Among all interventions, the highest SUCRA rankings were SEC-300 mg (86.01%), IXE-150 mg (76.91%), and BRO-210 mg (72.53%). Comparing different doses of highly ranked interventions, BRO-140 mg [RR = 2.61, 95%CrI = (1.38, 5.56)] and BRO-210 mg [RR = 3.47, 95%CrI = (1.89, 7.34)] significantly increased the number of patients achieving skin clearance compared to BRO-70 mg. Conversely, SEC-150 mg significantly reduced skin clearance compared to SEC-300 mg [RR = 0.53, 95%CrI = (0.38, 0.70)]. SUCRA rankings confirmed that BRO-210 mg ranked higher than BRO (70 mg, 140 mg), and SEC-300 mg ranked higher than SEC-150 mg. For lower-ranked doses, GUS-50 mg (68.52%) ranked higher than GUS (5 mg, 15 mg, 100 mg, 200 mg), TIL-200 mg (26.72%) ranked higher than TIL-100 mg, and UST-90 mg (45.82%) ranked higher than UST-45 mg (Tables 6, 7).

#### 3.4.4 Meta-analysis results of DLQI

**Figure 6:**
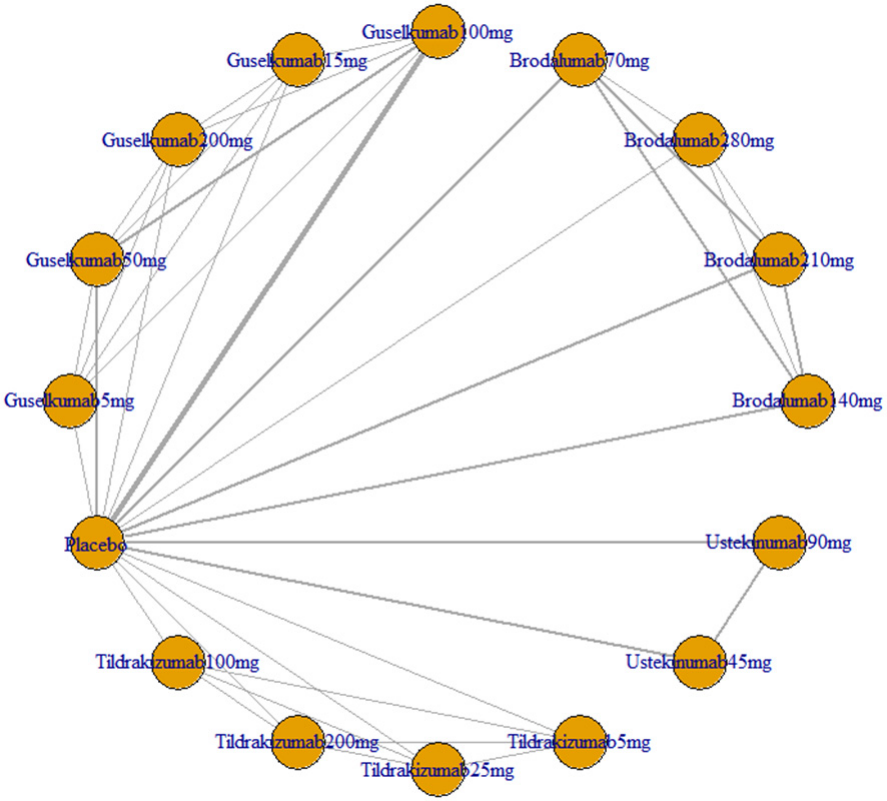
DLQI meta-network diagram.

For the DLQI outcome, a network meta-analysis 9 studies (11, 16, 17, 19–23, 27) involving 16 interventions and 4,337 patients from 9 RCTs was conducted, including 3,040 in the treatment groups and 1,297 in the placebo groups (Figure 6). Results showed no statistically significant differences between BRO-70 mg and placebo, while other IL-23/IL-17 inhibitors showed significant improvements in quality of life compared to placebo. The highest SUCRA rankings were TIL-25 mg (86.11%), TIL-200 mg (81.37%), and TIL-100 mg (77.10%). Comparisons of different doses of TIL showed that TIL-100 mg [MD = −3.59, 95%CrI (−6.86, −0.29)], TIL-200mg [MD = −3.9, 95%CrI (−7.18, −0.6)], and TIL-25 mg [MD = −4.27, 95%CrI (−7.58, −0.99)] significantly improved quality of life compared to TIL-5 mg. A reduction of this magnitude suggests clinically relevant improvement in patient-reported quality of life. SUCRA values confirmed that TIL-5 mg ranked lower than TIL-25 mg, TIL-100 mg, and TIL-200 mg, indicating that several TIL dose regimens ranked higher than TIL-5 mg in improving quality of life. Among other interventions, regimens with higher SUCRA rankings included BRO-210 mg (40.24%), which ranked higher than BRO-70 mg, BRO-140 mg, and BRO-280 mg; GUS-100 mg (75.50%), which ranked higher than GUS-5 mg, GUS-15 mg, GUS-50 mg, and GUS-200 mg; and UST-90 mg (71.00%), which ranked higher than UST-45 mg (Tables 8, 9).

**Table 8:**
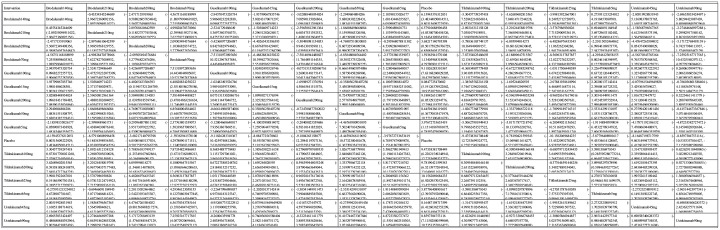
DLQI network meta-analysis results.

**Table 9:** Results of SUCRA of DLQI.

Brodalumab 140 mg	Brodalumab 210 mg	Brodalumab 280 mg
0.35622300	0.40242633	0.17764333
Brodalumab 70 mg	Guselkumab 100 mg	Guselkumab 15 mg
0.07451167	0.75504100	0.58280600
Guselkumab 200 mg	Guselkumab 50 mg	Guselkumab 5 mg
0.74323567	0.63468333	0.19275233
Placebo	Tildrakizumab 100 mg	Tildrakizumab 200 mg
0.00923900	0.77103200	0.81370300
Tildrakizumab 25 mg	Tildrakizumab 5 mg	Ustekinumab 45 mg
0.86112167	0.33650467	0.57911033
Ustekinumab 90 mg		
0.70996667		

#### 3.4.5 Descriptive assessment of ranking stability over time

Ranking stability over time was descriptively assessed by comparing the SUCRA rankings within each outcome across follow-up time points. SUCRA values were interpreted only within the same outcome and time point and were not directly compared across different outcomes or follow-up durations. Overall, treatment rankings varied over time. For PASI 75, SEC-300 mg and UST-90 mg ranked highly at early time points, whereas NET-120 mg, GUS-200 mg, and UST-90 mg ranked highly at week 12, and IXE regimens ranked highly at week 16. For PASI 90, BRO-210 mg showed relatively stable high rankings from weeks 4 to 12, while TIL-200 mg and GUS regimens ranked higher at week 16. For cleared or almost cleared skin, SEC-300 mg and BRO-210 mg showed favorable rankings across several early time points, whereas GUS-200 mg ranked highest at week 12. These findings indicate that no single regimen showed uniform superiority across all outcomes and time points; therefore, SUCRA rankings should be interpreted in an outcome- and time-specific manner.

#### 3.4.6 Adverse events (AEs) meta-analysis results

At 12 weeks of follow-up, a network meta-analysis was performed on 11 interventions across 13 RCTs involving 9,715 patients, with 7,129 in the treatment groups and 2,586 in the placebo groups. According to the league-table estimates, only BRO-140 mg, BRO-210 mg, SEC-150 mg, and SEC-300 mg showed a statistically significant increase in the risk of AEs compared with placebo. The interventions with the lowest SUCRA rankings were SEC-150 mg, BRO-210 mg, and SEC-300 mg. For upper respiratory tract infections, only SEC-150 mg and SEC-300 mg showed a significant increase in risk compared with placebo. Regarding discontinuation due to AEs, the risk was significantly higher for UST-45 mg than for placebo [RR = 7.81, 95% CrI = 2.00–60.98]. For SAEs, headache, and nasopharyngitis, the league tables showed no statistically significant differences between IL-23/IL-17 inhibitors and placebo. Detailed RR and 95% CrI estimates for all safety comparisons are provided in Supplementary materials 3, 4. SUCRA rankings were interpreted together with these effect estimates rather than as standalone evidence.

At 16 weeks of follow-up, a network meta-analysis was conducted on 7 interventions across 5 RCTs involving 1,726 patients, with 1,234 in the treatment groups and 492 in the placebo groups. For both AEs and SAEs, the league tables showed no statistically significant differences between the IL-23/IL-17 inhibitors and placebo. For AEs, the interventions with the lowest SUCRA rankings were GUS-100 mg and TIL-5 mg. For SAEs, the lowest SUCRA rankings were GUS-100 mg and TIL-200 mg. Although certain agents showed statistically increased risks of adverse events, the magnitude of risk elevation was generally modest, and serious adverse events did not differ significantly from placebo across most comparisons. Detailed league-table estimates, including RRs and 95% CrIs for all safety comparisons, are provided in Supplementary materials 3, 4.

### 3.5 Sensitivity analyses, publication bias and inconsistency

Sensitivity analyses excluding studies with high risk of bias were performed where the treatment network remained connected. The results were generally consistent with the primary analysis, suggesting that the main findings were not materially driven by studies at high risk of bias. Detailed results are presented in Supplementary material 5. Publication bias and small-study effects were assessed visually using funnel plots. Visual inspection of the funnel plots did not suggest obvious asymmetry for the main efficacy and safety outcomes. The corresponding funnel plots are provided in Supplementary material 6: Supplementary Figure S1 for PASI 75, Supplementary Figure S2 for PASI 90, Supplementary Figure S3 for cleared or almost cleared skin, Supplementary Figure S4 for DLQI, and Supplementary Figure S5 for adverse events. Egger's test was not performed because most direct pairwise comparisons included fewer than 10 studies. Node-splitting analysis could not be reliably performed for several outcomes because the network contained limited closed loops, and many comparisons were not informed by both direct and indirect evidence. This indicates sparsity of the treatment network, particularly for dose-specific nodes.

## 4 Discussion

Using a network meta-analysis, this study systematically evaluated the efficacy and safety of various IL-23/IL-17 inhibitors compared to placebo, covering multiple clinical outcome indicators such as PASI 75, PASI 90, complete skin clearance, DLQI, and adverse events (AEs). Comparing the effects of different treatment regimens, it further analyzed the impact of different dosages on efficacy and safety, providing strong evidence to support clinical practice. The results demonstrated that various IL-23/IL-17 inhibitors exhibited good efficacy in treating moderate-to-severe plaque psoriasis, especially in improving skin lesions and quality of life, with some higher-dose regimens showing stronger estimated effects in specific comparisons. Although SUCRA values provide a useful probabilistic ranking of treatments, they do not directly reflect the magnitude of clinical benefit. In some comparisons, treatments with similar effect sizes may receive different rankings due to small differences in estimated probabilities. Importantly, SUCRA values should not be directly compared across different outcomes or follow-up time points. A high ranking at an early induction time point does not necessarily indicate sustained superiority at later assessments. Therefore, treatment effectiveness should be interpreted within each prespecified outcome and follow-up duration, and in conjunction with the estimated effect sizes, 95% credible intervals, safety profiles, and the amount of evidence supporting each regimen.

Clinical interpretation of relative treatment effects also requires consideration of absolute benefit. PASI 75 and PASI 90 are established binary responder thresholds in psoriasis trials and therefore differ from continuous outcomes for which a single MCID can be directly applied. Achieving PASI 75 or PASI 90 itself reflects a clinically meaningful improvement in skin lesions; however, the clinical importance of a relative effect estimate may still depend on the baseline response probability. Therefore, RRs should be interpreted together with absolute response probabilities, placebo response rates, 95% credible intervals, and safety outcomes. A statistically significant RR may correspond to only a modest absolute benefit when the baseline response rate is low, whereas a similar RR may represent a larger absolute benefit when the baseline response rate is higher.

Our findings also need to be interpreted in relation to previous evidence. Erichsen et al. (6) reported that IL-17 inhibitors appeared more effective than IL-23 inhibitors but were associated with higher risks of adverse events. The present network meta-analysis partly supports this conclusion. Several IL-17 inhibitor regimens, particularly secukinumab- and brodalumab-based regimens, showed favorable short-term efficacy rankings for PASI 75 and PASI 90, and some of these regimens were also associated with increased risks of AEs or upper respiratory tract infections compared with placebo. However, our results also refine the conclusions of Erichsen et al. (6) by showing that class-level interpretation may oversimplify clinically relevant differences between individual agents, doses, outcomes, and follow-up durations. For example, GUS-200 mg ranked highly for PASI responses at several time points, and TIL-100 mg and TIL-200 mg showed favorable performance in skin clearance and DLQI improvement. Therefore, the comparative efficacy and safety of IL-17 and IL-23 inhibitors should not be interpreted solely at the drug-class level, but should be considered according to specific regimen, treatment goal, follow-up duration, and patient safety profile.

In the results of PASI 75 and PASI 90, significant differences in efficacy between different drugs were observed. Specifically, medications such as GUS-200 mg, SEC-300 mg, and BRO-210 mg frequently ranked among the higher-performing treatments according to SUCRA values; however, these rankings should be interpreted alongside the magnitude of treatment effects and their credible intervals. This finding is consistent with existing literature (31, 32). IL-23/IL-17 inhibitors enhance efficacy by specifically targeting T-cell activation and modulating immune responses (33). Among these inhibitors, Guselkumab and Secukinumab stand out. Research has shown that the IL-23/IL-17 pathway plays a crucial role in the pathogenesis of psoriasis, and these drugs effectively inhibit this pathway, leading to significant reductions in skin inflammation and hyperkeratosis (34, 35). Moreover, in some within-agent comparisons, certain higher-dose regimens showed stronger estimated effects than lower-dose regimens. However, because formal dose-response modeling was not performed and several dose-specific nodes were supported by limited evidence, these patterns should be interpreted as descriptive regimen-specific findings rather than statistically confirmed dose-response trends.

In terms of improving skin lesions and quality of life, this study also found that drugs such as TIL-100 mg and TIL-200 mg exhibited good efficacy, particularly at the 12-week follow-up, where TIL drugs showed significant improvements in quality of life. The improvement in quality of life with these drugs may be closely related to their significant clinical efficacy, as the improvement in skin lesions directly impacts patients' self-perception and social interactions. Improvements in DLQI are likely secondary to sustained skin clearance, reflecting the strong correlation between objective disease control and patient-reported outcomes (36). IL-17/IL-23 inhibitors not only effectively alleviate skin symptoms but also significantly enhance the daily quality of life for psoriasis patients (37, 38).

Regarding the occurrence of adverse events, we found that certain drugs (such as BRO-140 mg and SEC-150 mg) had a significantly higher incidence of adverse events compared to placebo, particularly with a marked increase in the occurrence of upper respiratory tract infections. This finding has been reported in several studies, suggesting that IL-23/IL-17 inhibitors may trigger abnormal immune responses or increase susceptibility to infections in some patients (6, 39). While IL-23/IL-17 inhibitors effectively treat inflammation by suppressing immune responses, they may also lower patients' immune defense functions, thereby increasing the risk of infections. Variations in immune status, underlying medical history, and the use of other immunosuppressive drugs in different patients could lead to individual differences in adverse events, which may explain why some drugs perform poorly in terms of adverse reactions. Although short-term adverse events were generally manageable across treatment groups, interpretation must remain cautious. IL-17 inhibitors have been associated with increased mucocutaneous candidiasis and potential inflammatory bowel disease signals, while IL-23 pathway inhibitors may carry infection-related risks. Given that most included trials were short-term induction studies, long-term safety, rare adverse events, and real-world pharmacovigilance signals could not be fully assessed in this analysis.

Given that psoriasis is a chronic, relapsing disease requiring sustained management, clinicians should interpret short-term efficacy rankings within the broader context of long-term therapeutic goals. While rapid and substantial PASI responses are desirable during induction, long-term treatment selection should balance efficacy with cumulative safety, durability of response, patient comorbidities, and real-world evidence. Short-term increases in adverse events do not necessarily predict long-term harm; however, they warrant careful monitoring, particularly when long-term safety data remain limited. Therefore, treatment decisions should integrate both the magnitude of short-term efficacy and the strength of long-term safety evidence. From a clinical perspective, the present findings may help inform treatment selection according to therapeutic goals rather than providing definitive phenotype-specific recommendations. When rapid short-term improvement in PASI 75 or PASI 90 is prioritized, regimens such as GUS-200 mg, SEC-300 mg, and BRO-210 mg may deserve attention because they frequently ranked highly at specific follow-up time points. When improvement in patient-reported quality of life or skin clearance is a major goal, TIL-100 mg and TIL-200 mg may be clinically relevant options based on their favorable performance in DLQI and cleared or almost cleared skin outcomes. However, for patients with greater concern about adverse events or infection-related risks, safety signals observed for some BRO- and SEC-based regimens should be carefully considered. Because the current evidence network was largely placebo-centered and contained limited head-to-head comparisons, these findings should be regarded as supportive evidence for individualized decision-making rather than definitive treatment recommendations.

These findings are broadly consistent with current clinical guidance, which recognizes IL-17 and IL-23 inhibitors as important biologic options for moderate-to-severe plaque psoriasis, particularly when rapid and substantial skin clearance is prioritized. Nevertheless, several limitations should be considered when interpreting the present findings. First, clinical and methodological heterogeneity existed across the included trials, including differences in patient populations, dose regimens, disease severity, and follow-up duration. These factors may have influenced the estimated efficacy and safety profiles of individual interventions. Second, safety outcomes were not uniformly reported across all trials. According to the risk-of-bias assessment, Reich et al. (27) was judged to have high risk of bias in selective reporting for adverse-event outcomes, and Nakagawa et al. (17) was assessed as having unclear risk in this domain. Therefore, safety signals, particularly those involving brodalumab and secukinumab, should be interpreted cautiously. Third, the treatment network was largely placebo-centered, with limited direct head-to-head comparisons between active biologics. Several treatment-dose nodes were supported by only one or a small number of trials, and some outcome-specific networks contained few or no closed loops. As a result, formal node-splitting analysis was not feasible for all outcomes, and inconsistency could not be fully evaluated across the entire network. This sparse evidence structure may reduce the precision of some estimates and the stability of SUCRA rankings, especially for dose-specific regimens. Therefore, treatment rankings should be interpreted as probabilistic estimates rather than definitive evidence of superiority and should be considered together with effect sizes, 95% credible intervals, absolute clinical relevance, and the amount of evidence supporting each comparison. Fourth, most included trials were short-term induction studies, with follow-up periods mainly ranging from 12 to 16 weeks. Although subgroup analyses by follow-up time point were conducted, the durability of response, long-term safety, rare adverse events, and real-world pharmacovigilance signals could not be fully assessed. Caution is therefore warranted when extrapolating these findings to long-term clinical management. Fifth, the search was restricted to English-language publications, which may introduce language bias. Although major biologic trials are usually published in international English-language journals, relevant studies published in other languages may have been missed. In addition, absolute risk differences were not used as the primary effect measure. Although PASI 75 and PASI 90 are clinically meaningful responder thresholds, reporting absolute response probabilities or risk differences alongside RRs may further improve clinical interpretability. Finally, the dose-specific node definition allowed regimen-level comparisons but increased network fragmentation. Formal dose-response or class-level analyses were beyond the scope of this prespecified regimen-level network meta-analysis. Therefore, the dose-related patterns observed in this study should be interpreted as descriptive regimen-specific findings rather than statistically confirmed dose-response trends. Future well-designed head-to-head randomized trials and long-term real-world studies are needed to confirm the comparative effectiveness, safety, and durability of these biologic regimens.

## 5 Conclusion

This Bayesian network meta-analysis provides a comparative, regimen-specific evaluation of the short-term efficacy and safety of IL-23 and IL-17 inhibitors for moderate-to-severe plaque psoriasis. Several regimens, including GUS-200 mg, SEC-300 mg, and BRO-210 mg, ranked highly for PASI 75 or PASI 90 at specific follow-up time points, whereas TIL-100 mg and TIL-200 mg showed favorable performance in quality-of-life and skin-clearance outcomes. These findings may help clinicians consider treatment options according to therapeutic goals, such as rapid skin response, quality-of-life improvement, or safety concerns. However, treatment rankings were largely informed by indirect comparisons within a predominantly placebo-centered network, and SUCRA values should be interpreted as probabilistic hierarchies rather than definitive evidence of superiority. Further long-term and head-to-head randomized trials are needed to confirm the comparative effectiveness, safety, and durability of these regimens.

## Data Availability

The original contributions presented in the study are included in the article/Supplementary material, further inquiries can be directed to the corresponding authors.
