# Supplementary material for: Efficacy and safety of interleukin inhibitors in the treatment of moderate-to-severe plaque psoriasis: a systematic review and network meta-analysis
Source: Front Med (Lausanne). 2026 Jul 23;13:1766348. doi: 10.3389/fmed.2026.1766348 (PMC13445711; doi:10.3389/fmed.2026.1766348)
Supplement: Supplementary file 1 [file Supplementary_file_1.docx]

**Pubmed**

| Search number | Query | Search Details | Results |
| --- | --- | --- | --- |
| 1 | ((((((((((((((((((((((((((((((((((((((((((((((((((((((((((((((((((((((((((((((Interleukins[MeSH Terms]) OR (Interleukins[Title/Abstract])) OR (Interleukin[Title/Abstract])) OR (interleukin derivative[Title/Abstract])) OR (brodalumab [Supplementary Concept])) OR (brodalumab[Title/Abstract])) OR (Siliq[Title/Abstract])) OR (amg827[Title/Abstract])) OR (khk4827[Title/Abstract])) OR (kyntheum[Title/Abstract])) OR (lumicef[Title/Abstract])) OR (ixekizumab [Supplementary Concept])) OR (ixekizumab[Title/Abstract])) OR (Taltz[Title/Abstract])) OR (ly2439821[Title/Abstract])) OR (secukinumab [Supplementary Concept])) OR (secukinumab[Title/Abstract])) OR (AIN457[Title/Abstract])) OR (Cosentyx[Title/Abstract])) OR (bat2306[Title/Abstract])) OR (cmab015[Title/Abstract])) OR (kb03303a[Title/Abstract])) OR (nvpain457[Title/Abstract])) OR (scapho[Title/Abstract])) OR (ts1808[Title/Abstract])) OR (bimekizumab [Supplementary Concept])) OR (bimekizumab[Title/Abstract])) OR (bimzelx[Title/Abstract])) OR (cdp4940[Title/Abstract])) OR (ucb4940[Title/Abstract])) OR (guselkumab [Supplementary Concept])) OR (guselkumab[Title/Abstract])) OR (Tremfya[Title/Abstract])) OR (cnto1959[Title/Abstract])) OR (risankizumab [Supplementary Concept])) OR (risankizumab[Title/Abstract])) OR (skyrizi[Title/Abstract])) OR (abbv066[Title/Abstract])) OR (bi655066[Title/Abstract])) OR (risankizumab rzaa[Title/Abstract])) OR (tildrakizumab [Supplementary Concept])) OR (tildrakizumab[Title/Abstract])) OR (Ilumya[Title/Abstract])) OR (ilumetri[Title/Abstract])) OR (mk3222[Title/Abstract])) OR (sch900222[Title/Abstract])) OR (sunpg1622[Title/Abstract])) OR (sunpg1623[Title/Abstract])) OR (Ustekinumab[MeSH Terms])) OR (Ustekinumab[Title/Abstract])) OR (Stelara[Title/Abstract])) OR (abp654[Title/Abstract])) OR (avt04[Title/Abstract])) OR (bat2206[Title/Abstract])) OR (bfi751[Title/Abstract])) OR (bmab1200[Title/Abstract])) OR (bow090[Title/Abstract])) OR (cnto1275[Title/Abstract])) OR (ctp43[Title/Abstract])) OR (dmb3115[Title/Abstract])) OR (eb1004[Title/Abstract])) OR (fyb202[Title/Abstract])) OR (monoclonal antibody cnto 1275[Title/Abstract])) OR (ons3040[Title/Abstract])) OR (pb007[Title/Abstract])) OR (ro7233920[Title/Abstract])) OR (sb17[Title/Abstract])) OR (stellara[Title/Abstract])) OR (netakimab [Supplementary Concept])) OR (netakimab[Title/Abstract])) OR (mirikizumab [Supplementary Concept])) OR (mirikizumab[Title/Abstract])) OR (LY3074828[Title/Abstract])) OR (omvoh[Title/Abstract])) OR (Izokibep[Title/Abstract])) OR (aby035[Title/Abstract])) OR (afb035[Title/Abstract])) OR (img020[Title/Abstract])) OR (HB0017[Title/Abstract]) | "Interleukins"[MeSH Terms] OR "Interleukins"[Title/Abstract] OR "Interleukin"[Title/Abstract] OR (("interleukine"[All Fields] OR "interleukines"[All Fields] OR "Interleukins"[MeSH Terms] OR "Interleukins"[All Fields] OR "Interleukin"[All Fields]) AND "derivative"[Title/Abstract]) OR "brodalumab"[Supplementary Concept] OR "brodalumab"[Title/Abstract] OR "Siliq"[Title/Abstract] OR "amg827"[Title/Abstract] OR "khk4827"[Title/Abstract] OR "kyntheum"[Title/Abstract] OR "lumicef"[Title/Abstract] OR "ixekizumab"[Supplementary Concept] OR "ixekizumab"[Title/Abstract] OR "Taltz"[Title/Abstract] OR "ly2439821"[Title/Abstract] OR "secukinumab"[Supplementary Concept] OR "secukinumab"[Title/Abstract] OR "AIN457"[Title/Abstract] OR "Cosentyx"[Title/Abstract] OR "scapho"[Title/Abstract] OR "ts1808"[Title/Abstract] OR "bimekizumab"[Supplementary Concept] OR "bimekizumab"[Title/Abstract] OR "bimzelx"[Title/Abstract] OR "ucb4940"[Title/Abstract] OR "guselkumab"[Supplementary Concept] OR "guselkumab"[Title/Abstract] OR "Tremfya"[Title/Abstract] OR "cnto1959"[Title/Abstract] OR "risankizumab"[Supplementary Concept] OR "risankizumab"[Title/Abstract] OR "skyrizi"[Title/Abstract] OR "abbv066"[Title/Abstract] OR "bi655066"[Title/Abstract] OR "risankizumab rzaa"[Title/Abstract] OR "tildrakizumab"[Supplementary Concept] OR "tildrakizumab"[Title/Abstract] OR "Ilumya"[Title/Abstract] OR "ilumetri"[Title/Abstract] OR "mk3222"[Title/Abstract] OR "Ustekinumab"[MeSH Terms] OR "Ustekinumab"[Title/Abstract] OR "Stelara"[Title/Abstract] OR "avt04"[Title/Abstract] OR "bat2206"[Title/Abstract] OR "bfi751"[Title/Abstract] OR "cnto1275"[Title/Abstract] OR "ctp43"[Title/Abstract] OR (("antibodies, monoclonal"[MeSH Terms] OR ("antibodies"[All Fields] AND "monoclonal"[All Fields]) OR "monoclonal antibodies"[All Fields] OR ("monoclonal"[All Fields] AND "antibody"[All Fields]) OR "monoclonal antibody"[All Fields]) AND "cnto 1275"[Title/Abstract]) OR "sb17"[Title/Abstract] OR "netakimab"[Supplementary Concept] OR "netakimab"[Title/Abstract] OR "mirikizumab"[Supplementary Concept] OR "mirikizumab"[Title/Abstract] OR "LY3074828"[Title/Abstract] OR "omvoh"[Title/Abstract] OR "Izokibep"[Title/Abstract] OR "aby035"[Title/Abstract] OR "HB0017"[Title/Abstract] | 417,275 |
| 2 | (((((((((((((((Psoriasis[MeSH Terms]) OR (Psoriasis[Title/Abstract])) OR (Psoriases[Title/Abstract])) OR (Pustulosis of Palms[Title/Abstract] AND Soles[Title/Abstract])) OR (Pustulosis Palmaris et Plantaris[Title/Abstract])) OR (Palmoplantaris Pustulosis[Title/Abstract])) OR (Pustular Psoriasis of Palms[Title/Abstract] AND Soles[Title/Abstract])) OR (psoriasiform dermatitis[Title/Abstract])) OR (psoriasiform dermatosis[Title/Abstract])) OR (psoriasiform lesion[Title/Abstract])) OR (psoriasiform rash[Title/Abstract])) OR (psoriasiform skin rash[Title/Abstract])) OR (psoriatic epidermis[Title/Abstract])) OR (psoriatic skin[Title/Abstract])) OR (skin rash, psoriasiform[Title/Abstract])) OR (willan lepra[Title/Abstract]) | "Psoriasis"[MeSH Terms] OR "Psoriasis"[Title/Abstract] OR "Psoriases"[Title/Abstract] OR ("Pustulosis"[All Fields] AND "of palms"[Title/Abstract] AND "Soles"[Title/Abstract]) OR "pustulosis palmaris et plantaris"[Title/Abstract] OR "palmoplantaris pustulosis"[Title/Abstract] OR ("pustular psoriasis of palms"[Title/Abstract] AND "Soles"[Title/Abstract]) OR "psoriasiform dermatitis"[Title/Abstract] OR "psoriasiform dermatosis"[Title/Abstract] OR "psoriasiform lesion"[Title/Abstract] OR "psoriasiform rash"[Title/Abstract] OR "psoriasiform skin rash"[Title/Abstract] OR "psoriatic epidermis"[Title/Abstract] OR "psoriatic skin"[Title/Abstract] OR (("exanthema"[MeSH Terms] OR "exanthema"[All Fields] OR ("skin"[All Fields] AND "rash"[All Fields]) OR "skin rash"[All Fields]) AND "psoriasiform"[Title/Abstract]) OR (("willan"[All Fields] OR "willan s"[All Fields]) AND "lepra"[Title/Abstract]) | 65,913 |
| 3 | ((((((((((((((((Psoriasis[MeSH Terms]) OR (Psoriasis[Title/Abstract])) OR (Psoriases[Title/Abstract])) OR (Pustulosis of Palms[Title/Abstract] AND Soles[Title/Abstract])) OR (Pustulosis Palmaris et Plantaris[Title/Abstract])) OR (Palmoplantaris Pustulosis[Title/Abstract])) OR (Pustular Psoriasis of Palms[Title/Abstract] AND Soles[Title/Abstract])) OR (psoriasiform dermatitis[Title/Abstract])) OR (psoriasiform dermatosis[Title/Abstract])) OR (psoriasiform lesion[Title/Abstract])) OR (psoriasiform rash[Title/Abstract])) OR (psoriasiform skin rash[Title/Abstract])) OR (psoriatic epidermis[Title/Abstract])) OR (psoriatic skin[Title/Abstract])) OR (skin rash, psoriasiform[Title/Abstract])) OR (willan lepra[Title/Abstract])) AND (((((((((((((((((((((((((((((((((((((((((((((((((((((((((((((((((((((((((((((((Interleukins[MeSH Terms]) OR (Interleukins[Title/Abstract])) OR (Interleukin[Title/Abstract])) OR (interleukin derivative[Title/Abstract])) OR (brodalumab [Supplementary Concept])) OR (brodalumab[Title/Abstract])) OR (Siliq[Title/Abstract])) OR (amg827[Title/Abstract])) OR (khk4827[Title/Abstract])) OR (kyntheum[Title/Abstract])) OR (lumicef[Title/Abstract])) OR (ixekizumab [Supplementary Concept])) OR (ixekizumab[Title/Abstract])) OR (Taltz[Title/Abstract])) OR (ly2439821[Title/Abstract])) OR (secukinumab [Supplementary Concept])) OR (secukinumab[Title/Abstract])) OR (AIN457[Title/Abstract])) OR (Cosentyx[Title/Abstract])) OR (bat2306[Title/Abstract])) OR (cmab015[Title/Abstract])) OR (kb03303a[Title/Abstract])) OR (nvpain457[Title/Abstract])) OR (scapho[Title/Abstract])) OR (ts1808[Title/Abstract])) OR (bimekizumab [Supplementary Concept])) OR (bimekizumab[Title/Abstract])) OR (bimzelx[Title/Abstract])) OR (cdp4940[Title/Abstract])) OR (ucb4940[Title/Abstract])) OR (guselkumab [Supplementary Concept])) OR (guselkumab[Title/Abstract])) OR (Tremfya[Title/Abstract])) OR (cnto1959[Title/Abstract])) OR (risankizumab [Supplementary Concept])) OR (risankizumab[Title/Abstract])) OR (skyrizi[Title/Abstract])) OR (abbv066[Title/Abstract])) OR (bi655066[Title/Abstract])) OR (risankizumab rzaa[Title/Abstract])) OR (tildrakizumab [Supplementary Concept])) OR (tildrakizumab[Title/Abstract])) OR (Ilumya[Title/Abstract])) OR (ilumetri[Title/Abstract])) OR (mk3222[Title/Abstract])) OR (sch900222[Title/Abstract])) OR (sunpg1622[Title/Abstract])) OR (sunpg1623[Title/Abstract])) OR (Ustekinumab[MeSH Terms])) OR (Ustekinumab[Title/Abstract])) OR (Stelara[Title/Abstract])) OR (abp654[Title/Abstract])) OR (avt04[Title/Abstract])) OR (bat2206[Title/Abstract])) OR (bfi751[Title/Abstract])) OR (bmab1200[Title/Abstract])) OR (bow090[Title/Abstract])) OR (cnto1275[Title/Abstract])) OR (ctp43[Title/Abstract])) OR (dmb3115[Title/Abstract])) OR (eb1004[Title/Abstract])) OR (fyb202[Title/Abstract])) OR (monoclonal antibody cnto 1275[Title/Abstract])) OR (ons3040[Title/Abstract])) OR (pb007[Title/Abstract])) OR (ro7233920[Title/Abstract])) OR (sb17[Title/Abstract])) OR (stellara[Title/Abstract])) OR (netakimab [Supplementary Concept])) OR (netakimab[Title/Abstract])) OR (mirikizumab [Supplementary Concept])) OR (mirikizumab[Title/Abstract])) OR (LY3074828[Title/Abstract])) OR (omvoh[Title/Abstract])) OR (Izokibep[Title/Abstract])) OR (aby035[Title/Abstract])) OR (afb035[Title/Abstract])) OR (img020[Title/Abstract])) OR (HB0017[Title/Abstract])) | ("Psoriasis"[MeSH Terms] OR "Psoriasis"[Title/Abstract] OR "Psoriases"[Title/Abstract] OR ("Pustulosis"[All Fields] AND "of palms"[Title/Abstract] AND "Soles"[Title/Abstract]) OR "pustulosis palmaris et plantaris"[Title/Abstract] OR "palmoplantaris pustulosis"[Title/Abstract] OR ("pustular psoriasis of palms"[Title/Abstract] AND "Soles"[Title/Abstract]) OR "psoriasiform dermatitis"[Title/Abstract] OR "psoriasiform dermatosis"[Title/Abstract] OR "psoriasiform lesion"[Title/Abstract] OR "psoriasiform rash"[Title/Abstract] OR "psoriasiform skin rash"[Title/Abstract] OR "psoriatic epidermis"[Title/Abstract] OR "psoriatic skin"[Title/Abstract] OR (("exanthema"[MeSH Terms] OR "exanthema"[All Fields] OR ("skin"[All Fields] AND "rash"[All Fields]) OR "skin rash"[All Fields]) AND "psoriasiform"[Title/Abstract]) OR (("willan"[All Fields] OR "willan s"[All Fields]) AND "lepra"[Title/Abstract])) AND ("Interleukins"[MeSH Terms] OR "Interleukins"[Title/Abstract] OR "Interleukin"[Title/Abstract] OR (("interleukine"[All Fields] OR "interleukines"[All Fields] OR "Interleukins"[MeSH Terms] OR "Interleukins"[All Fields] OR "Interleukin"[All Fields]) AND "derivative"[Title/Abstract]) OR "brodalumab"[Supplementary Concept] OR "brodalumab"[Title/Abstract] OR "Siliq"[Title/Abstract] OR "amg827"[Title/Abstract] OR "khk4827"[Title/Abstract] OR "kyntheum"[Title/Abstract] OR "lumicef"[Title/Abstract] OR "ixekizumab"[Supplementary Concept] OR "ixekizumab"[Title/Abstract] OR "Taltz"[Title/Abstract] OR "ly2439821"[Title/Abstract] OR "secukinumab"[Supplementary Concept] OR "secukinumab"[Title/Abstract] OR "AIN457"[Title/Abstract] OR "Cosentyx"[Title/Abstract] OR "scapho"[Title/Abstract] OR "ts1808"[Title/Abstract] OR "bimekizumab"[Supplementary Concept] OR "bimekizumab"[Title/Abstract] OR "bimzelx"[Title/Abstract] OR "ucb4940"[Title/Abstract] OR "guselkumab"[Supplementary Concept] OR "guselkumab"[Title/Abstract] OR "Tremfya"[Title/Abstract] OR "cnto1959"[Title/Abstract] OR "risankizumab"[Supplementary Concept] OR "risankizumab"[Title/Abstract] OR "skyrizi"[Title/Abstract] OR "abbv066"[Title/Abstract] OR "bi655066"[Title/Abstract] OR "risankizumab rzaa"[Title/Abstract] OR "tildrakizumab"[Supplementary Concept] OR "tildrakizumab"[Title/Abstract] OR "Ilumya"[Title/Abstract] OR "ilumetri"[Title/Abstract] OR "mk3222"[Title/Abstract] OR "Ustekinumab"[MeSH Terms] OR "Ustekinumab"[Title/Abstract] OR "Stelara"[Title/Abstract] OR "avt04"[Title/Abstract] OR "bat2206"[Title/Abstract] OR "bfi751"[Title/Abstract] OR "cnto1275"[Title/Abstract] OR "ctp43"[Title/Abstract] OR (("antibodies, monoclonal"[MeSH Terms] OR ("antibodies"[All Fields] AND "monoclonal"[All Fields]) OR "monoclonal antibodies"[All Fields] OR ("monoclonal"[All Fields] AND "antibody"[All Fields]) OR "monoclonal antibody"[All Fields]) AND "cnto 1275"[Title/Abstract]) OR "sb17"[Title/Abstract] OR "netakimab"[Supplementary Concept] OR "netakimab"[Title/Abstract] OR "mirikizumab"[Supplementary Concept] OR "mirikizumab"[Title/Abstract] OR "LY3074828"[Title/Abstract] OR "omvoh"[Title/Abstract] OR "Izokibep"[Title/Abstract] OR "aby035"[Title/Abstract] OR "HB0017"[Title/Abstract]) | 8,997 |

**Cochrance**

| ID | Search | Hits |
| --- | --- | --- |
| #1 | MeSH descriptor: [Receptors, Interleukin] explode all trees | 1126 |
| #2 | (Interleukins):ti,ab,kw OR (Interleukin):ti,ab,kw OR (interleukin derivative):ti,ab,kw OR (brodalumab):ti,ab,kw OR (Siliq):ti,ab,kw | 25469 |
| #3 | (amg827):ti,ab,kw OR (khk4827):ti,ab,kw OR (kyntheum):ti,ab,kw OR (lumicef):ti,ab,kw OR (ixekizumab):ti,ab,kw | 683 |
| #4 | (Taltz):ti,ab,kw OR (ly2439821):ti,ab,kw OR (secukinumab):ti,ab,kw OR (AIN457):ti,ab,kw OR (Cosentyx):ti,ab,kw | 1246 |
| #5 | (bat2306):ti,ab,kw OR (cmab015):ti,ab,kw OR (kb03303a):ti,ab,kw OR (nvpain457):ti,ab,kw OR (scapho):ti,ab,kw | 10 |
| #6 | (ts1808):ti,ab,kw OR (bimekizumab):ti,ab,kw OR (bimzelx):ti,ab,kw OR (cdp4940):ti,ab,kw OR (ucb4940):ti,ab,kw | 250 |
| #7 | (guselkumab):ti,ab,kw OR (Tremfya):ti,ab,kw OR (cnto1959):ti,ab,kw OR (risankizumab):ti,ab,kw OR (skyrizi):ti,ab,kw | 916 |
| #8 | (abbv066):ti,ab,kw OR (bi655066):ti,ab,kw OR (risankizumab rzaa):ti,ab,kw OR (tildrakizumab):ti,ab,kw OR (Ilumya):ti,ab,kw | 204 |
| #9 | (ilumetri):ti,ab,kw OR (mk3222):ti,ab,kw OR (sch900222):ti,ab,kw OR (sunpg1622):ti,ab,kw OR (sunpg1623):ti,ab,kw | 4 |
| #10 | MeSH descriptor: [undefined] explode all trees | 0 |
| #11 | (Ustekinumab):ti,ab,kw OR (Stelara):ti,ab,kw OR (abp654):ti,ab,kw OR (avt04):ti,ab,kw OR (bat2206):ti,ab,kw | 1197 |
| #12 | (bfi751):ti,ab,kw OR (bmab1200):ti,ab,kw OR (bow090):ti,ab,kw OR (cnto1275):ti,ab,kw OR (ctp43):ti,ab,kw | 37 |
| #13 | (dmb3115):ti,ab,kw OR (eb1004):ti,ab,kw OR (fyb202):ti,ab,kw OR (monoclonal antibody cnto 1275):ti,ab,kw OR (ons3040):ti,ab,kw | 14 |
| #14 | (pb007):ti,ab,kw OR (ro7233920):ti,ab,kw OR (sb17):ti,ab,kw OR (stellara):ti,ab,kw OR (netakimab):ti,ab,kw | 27 |
| #15 | (mirikizumab):ti,ab,kw OR (LY3074828):ti,ab,kw OR (omvoh):ti,ab,kw OR (Izokibep):ti,ab,kw OR (aby035):ti,ab,kw | 214 |
| #16 | (afb035):ti,ab,kw OR (img020):ti,ab,kw OR (HB0017):ti,ab,kw | 4 |
| #17 | #1 or #2 or #3 or #4 or #5 or #6 or #7 or #8 or #9 or #10 or #11 or #12 or #13 or #14 or #15 or #16 | 28598 |
| #18 | MeSH descriptor: [Psoriasis] explode all trees | 4743 |
| #19 | (Psoriasis):ti,ab,kw OR (Psoriases):ti,ab,kw OR (Pustulosis of Palms and Soles):ti,ab,kw OR (Pustulosis Palmaris et Plantaris):ti,ab,kw OR (Palmoplantaris Pustulosis):ti,ab,kw | 10052 |
| #20 | (Pustular Psoriasis of Palms and Soles):ti,ab,kw OR (psoriasiform dermatitis):ti,ab,kw OR (psoriasiform dermatosis):ti,ab,kw OR (psoriasiform lesion):ti,ab,kw OR (psoriasiform rash):ti,ab,kw | 35 |
| #21 | (psoriasiform skin rash):ti,ab,kw OR (psoriatic epidermis):ti,ab,kw OR (psoriatic skin):ti,ab,kw OR (skin rash, psoriasiform):ti,ab,kw OR (willan lepra):ti,ab,kw | 1300 |
| #22 | #18 or #19 or #20 or #21 | 10635 |
| #23 | #17 and #22 | 2822 |

**Embase**

| No. | Query Results | Results |
| --- | --- | --- |
| #3. | #1 AND #2 | 17,912 |
| #2. | 'psoriasis'/exp OR psoriasis:ti,ab,kw OR | 129,546 |
|  | psoriases:ti,ab,kw OR ('pustulosis of |  |
|  | palms':ti,ab,kw AND soles:ti,ab,kw) OR |  |
|  | 'pustulosis palmaris et plantaris':ti,ab,kw OR |  |
|  | 'palmoplantaris pustulosis':ti,ab,kw OR |  |
|  | ('pustular psoriasis of palms':ti,ab,kw AND |  |
|  | soles:ti,ab,kw) OR 'psoriasiform |  |
|  | dermatitis':ti,ab,kw OR 'psoriasiform |  |
|  | dermatosis':ti,ab,kw OR 'psoriasiform |  |
|  | lesion':ti,ab,kw OR 'psoriasiform rash':ti,ab,kw |  |
|  | OR 'psoriasiform skin rash':ti,ab,kw OR |  |
|  | 'psoriatic epidermis':ti,ab,kw OR 'psoriatic |  |
|  | skin':ti,ab,kw OR 'skin rash, |  |
|  | psoriasiform':ti,ab,kw OR 'willan lepra':ti,ab,kw |  |
| #1. | 'interleukin derivative'/exp OR 'interleukin | 382,097 |
|  | derivative':ti,ab,kw OR interleukin:ti,ab,kw OR |  |
|  | interleukins:ti,ab,kw OR 'brodalumab'/exp OR |  |
|  | brodalumab:ti,ab,kw OR siliq:ti,ab,kw OR |  |
|  | amg827:ti,ab,kw OR khk4827:ti,ab,kw OR |  |
|  | kyntheum:ti,ab,kw OR lumicef:ti,ab,kw OR |  |
|  | 'ixekizumab'/exp OR ixekizumab:ti,ab,kw OR |  |
|  | taltz:ti,ab,kw OR ly2439821:ti,ab,kw OR |  |
|  | 'secukinumab'/exp OR secukinumab:ti,ab,kw OR |  |
|  | ain457:ti,ab,kw OR cosentyx:ti,ab,kw OR |  |
|  | bat2306:ti,ab,kw OR cmab015:ti,ab,kw OR |  |
|  | kb03303a:ti,ab,kw OR nvpain457:ti,ab,kw OR |  |
|  | scapho:ti,ab,kw OR ts1808:ti,ab,kw OR |  |
|  | 'bimekizumab'/exp OR bimekizumab:ti,ab,kw OR |  |
|  | bimzelx:ti,ab,kw OR cdp4940:ti,ab,kw OR |  |
|  | ucb4940:ti,ab,kw OR 'guselkumab'/exp OR |  |
|  | guselkumab:ti,ab,kw OR tremfya:ti,ab,kw OR |  |
|  | cnto1959:ti,ab,kw OR 'risankizumab'/exp OR |  |
|  | risankizumab:ti,ab,kw OR skyrizi:ti,ab,kw OR |  |
|  | abbv066:ti,ab,kw OR bi655066:ti,ab,kw OR |  |
|  | 'risankizumab rzaa':ti,ab,kw OR |  |
|  | 'tildrakizumab'/exp OR tildrakizumab:ti,ab,kw OR |  |
|  | ilumya:ti,ab,kw OR ilumetri:ti,ab,kw OR |  |
|  | mk3222:ti,ab,kw OR sch900222:ti,ab,kw OR |  |
|  | sunpg1622:ti,ab,kw OR sunpg1623:ti,ab,kw OR |  |
|  | 'ustekinumab'/exp OR ustekinumab:ti,ab,kw OR |  |
|  | stelara:ti,ab,kw OR abp654:ti,ab,kw OR |  |
|  | avt04:ti,ab,kw OR bat2206:ti,ab,kw OR |  |
|  | bfi751:ti,ab,kw OR bmab1200:ti,ab,kw OR |  |
|  | bow090:ti,ab,kw OR cnto1275:ti,ab,kw OR |  |
|  | ctp43:ti,ab,kw OR dmb3115:ti,ab,kw OR |  |
|  | eb1004:ti,ab,kw OR fyb202:ti,ab,kw OR 'monoclonal |  |
|  | antibody cnto 1275':ti,ab,kw OR ons3040:ti,ab,kw |  |
|  | OR pb007:ti,ab,kw OR ro7233920:ti,ab,kw OR |  |
|  | sb17:ti,ab,kw OR stellara:ti,ab,kw OR |  |
|  | 'netakimab'/exp OR netakimab:ti,ab,kw OR |  |
|  | 'mirikizumab'/exp OR mirikizumab:ti,ab,kw OR |  |
|  | ly3074828:ti,ab,kw OR omvoh:ti,ab,kw OR |  |
|  | 'izokibep'/exp OR izokibep:ti,ab,kw OR |  |
|  | aby035:ti,ab,kw OR afb035:ti,ab,kw OR |  |
|  | 'img020'/exp OR hb0017:ti,ab,kw |  |

**Wos**

| # Searches: |  |
| --- | --- |
|  |  |
| 1: Interleukins (Topic) OR Interleukin (Topic) OR interleukin derivative (Topic) OR brodalumab (Topic) OR Siliq (Topic) OR amg827 (Topic) OR khk4827 (Topic) OR kyntheum (Topic) OR lumicef (Topic) OR ixekizumab (Topic) OR Taltz (Topic) OR ly2439821 (Topic) OR secukinumab (Topic) OR AIN457 (Topic) OR Cosentyx (Topic) OR bat2306 (Topic) OR cmab015 (Topic) OR kb03303a (Topic) OR nvpain457 (Topic) OR scapho (Topic) OR ts1808 (Topic) OR bimekizumab (Topic) OR bimzelx (Topic) OR cdp4940 (Topic) OR ucb4940 (Topic) OR guselkumab (Topic) OR Tremfya (Topic) OR cnto1959 (Topic) OR risankizumab (Topic) OR skyrizi (Topic) OR abbv066 (Topic) OR bi655066 (Topic) OR risankizumab rzaa (Topic) OR tildrakizumab (Topic) OR Ilumya (Topic) OR ilumetri (Topic) OR mk3222 (Topic) OR sch900222 (Topic) OR sunpg1622 (Topic) OR sunpg1623 (Topic) OR Ustekinumab (Topic) OR Stelara (Topic) OR abp654 (Topic) OR avt04 (Topic) OR bat2206 (Topic) OR bfi751 (Topic) OR bmab1200 (Topic) OR bow090 (Topic) OR cnto1275 (Topic) OR ctp43 (Topic) OR dmb3115 (Topic) OR eb1004 (Topic) OR fyb202 (Topic) OR monoclonal antibody cnto 1275 (Topic) OR ons3040 (Topic) OR pb007 (Topic) OR ro7233920 (Topic) OR sb17 (Topic) OR stellara (Topic) OR netakimab (Topic) OR mirikizumab (Topic) OR LY3074828 (Topic) OR omvoh (Topic) OR Izokibep (Topic) OR aby035 (Topic) OR afb035 (Topic) OR img020 (Topic) OR HB0017 (Topic) | Results: 399765 |
|  |  |
| 2: Psoriasis (Topic) OR Psoriases (Topic) OR Pustulosis of Palms and Soles (Topic) OR brodalumabPustulosis Palmaris et Plantaris (Topic) OR Palmoplantaris Pustulosis (Topic) OR Pustular Psoriasis of Palms and Soles (Topic) OR psoriasiform dermatitis (Topic) OR psoriasiform dermatosis (Topic) OR psoriasiform lesion (Topic) OR psoriasiform rash (Topic) OR psoriasiform skin rash (Topic) OR psoriatic epidermis (Topic) OR psoriatic skin (Topic) OR skin rash, psoriasiform (Topic) OR willan lepra (Topic) | Results: 68213 |
|  |  |
| 3: #2 AND #1 | Results: 9941 |
|  |  |
| 4: #2 AND #1 | Results: 9941 |
