# Supplementary material for: Efficacy and safety of interleukin inhibitors in the treatment of moderate-to-severe plaque psoriasis: a systematic review and network meta-analysis
Source: Front Med (Lausanne). 2026 Jul 23;13:1766348. doi: 10.3389/fmed.2026.1766348 (PMC13445711; doi:10.3389/fmed.2026.1766348)
Supplement: Supplementary file 2 [file Supplementary_file_2.docx]

**Identification of studies via databases and registers (From 30 May 2024 to 25 April 2026)**

Records identified from:

PubMed (n =1407 )

Embase (n =2573 )

Cochrane (n =236 )

Web of Science (n =1370 )

Records removed before screening:

Duplicate records removed

(n = 867)

**Identification**

Records excluded (n = 4511):

Letters/notes/case report (n=223)

Non-RCT (n=2157)

Non-English (n=6)

Reviews/conference (n=1187)

Ineligible population (n=472)

Ineligible intervention (n=466)

Records screened (n = 4719)

**Screening**

Reports assessed for

eligibility (n = 208)

**Included**

Potentially relevant studies identified in the updated search (n = 6; not incorporated into the current primary quantitative synthesis)

Reports not retrieved

(n = 0)

Reports sought for

retrieval (n = 208)

Reports excluded (n = 202):

Cohort (n=39)

Non-RCT (n=79)

Mechanistic study (n=6)

Ineligible population (n=42)

Ineligible intervention (n=36)

**literature list**

**(Cestari, Souza et al. 2024, Papp, Lebwohl et al. 2024, Cai, Li et al. 2025, Gold, Armstrong et al. 2025, Papp, Balser et al. 2025, Cai, Wang et al. 2026)**

Cai, L., C. Li, S. Li, X. Zhang, G. Wang, J. Yu, K. Huang, H. Fang, Y. Ding, J. Wang, C. Jiang, Q. Lu, J. Tao and J. Zhang (2025). "Investigation of the Efficacy and Safety of Xeligekimab (GR1501) in Patients with Moderate-to-Severe Plaque Psoriasis: A Multicenter, Randomized, Double-Blind Phase II Clinical trial." Dermatology and Therapy **15**(10): 2803-2816.

Cai, L., X. Wang, L. Zhang, G. Zhang, L. Wu, B. Yang, Z. Meng, J. Shi, G. Han, A. Wei, S. Li, Y. Zhao, J. Su, N. Wei, S. Liu, R. Liu, W. Liu, M. Fang, X. Zhang and J. Zhang (2026). "HS-20137 for Chinese Moderate-to-Severe Psoriasis: A Randomized, Double-Blinded, Phase 2 Study." Dermatology and Therapy.

Cestari, T. F., C. d. S. Souza, L. Azulay-Abulafia, R. Romiti, A. V. E. Carvalho, C. C. Silva de Castro, S. A. Marques, J. R. Antonio, L. Fabrício, A. M. Soliman, T. Wu, R. Sinvhal, V. Stakias, A. P. Song, J. Kalabic, N. Martin and L. K. M. Oyafuso (2024). "Efficacy and safety of risankizumab versus methotrexate in patients with moderate-to-severe plaque psoriasis: results from IMMbrace, a randomized, double-blind, phase 3 study with an open-label extension period in Brazil." Anais Brasileiros de Dermatologia **100**(2): 260-271.

Gold, L. S., A. W. Armstrong, R. Bissonnette, N. Magnolo, R. B. Vender, M. Sebastian, M. L. Galimberti, A. Tsianakas, M. Arnone, P. Wallace, M. Simon, J. Riera-Monroig, S. Gerdes, J. Waibel, A. Gonzalez-Cantero, B. Schwarz, Y. Tada, M. Cecchini, B. Ehst, L. Kircik, L. Kephart, O. Reyes-Servin, B. E. Edem, J. H. Campbell, Y.-K. Shen, K. Cresswell, S. Li, C. M. C. DeKlotz, F. Nunes and K. A. Papp (2025). "Once-daily oral icotrokinra versus placebo and once-daily oral deucravacitinib in participants with moderate-to-severe plaque psoriasis (ICONIC-ADVANCE 1 &amp; 2): two phase 3, randomised, placebo-controlled and active-comparator-controlled trials." The Lancet **406**(10510): 1363-1374.

Papp, K., S. Balser, K. Nopora, P. Rewerski, B. Freudensprung and M. Trieb (2025). "A Randomised, Double-Blind Trial to Compare the Efficacy, Safety, and Immunogenicity of the Biosimilar Ustekinumab FYB202 with Reference Ustekinumab in Patients with Moderate-to-Severe Plaque Psoriasis." Advances in Therapy **42**(5): 2135-2149.

Papp, K. A., M. G. Lebwohl, D. Thaçi, J. Jaworski, B. Kwiek, J. Trefler, A. Dudek, J. C. Szepietowski, N. Reznichenko, J. Narbutt, W. Baran, J. Kolinek, S. Daniluk, K. Bartnicka-Maslowska, A. Reich, Y. Andrashko, S. Kim, Y. Bae, D. Jeon, J. Jung, H. Lee, T. Pyo and W. Ko (2024). "Efficacy and Safety of Candidate Biosimilar CT-P43 Versus Originator Ustekinumab in Moderate to Severe Plaque Psoriasis: 28-Week Results of a Randomised, Active-Controlled, Double-Blind, Phase III Study." BioDrugs **38**(1): 121-131.
