# Supplementary material for: Efficacy and safety of interleukin inhibitors in the treatment of moderate-to-severe plaque psoriasis: a systematic review and network meta-analysis
Source: Front Med (Lausanne). 2026 Jul 23;13:1766348. doi: 10.3389/fmed.2026.1766348 (PMC13445711; doi:10.3389/fmed.2026.1766348)
Supplement: Supplementary file 3 [file Supplementary_file_3.docx]

AE 12W meta-networ diagram

AE 16W meta-networ diagram

discontinuation 12W network meta-analysis

Headache 12W network meta-analysis

 Nasopharyngitis 12W network meta-analysis

 SAE 12W network meta-analysis

 SAE 16W network meta-analysis diagram

tract infection 12W network meta-analysis
