# Supplementary material for: Efficacy and safety of interleukin inhibitors in the treatment of moderate-to-severe plaque psoriasis: a systematic review and network meta-analysis
Source: Front Med (Lausanne). 2026 Jul 23;13:1766348. doi: 10.3389/fmed.2026.1766348 (PMC13445711; doi:10.3389/fmed.2026.1766348)
Supplement: Supplementary file 4 [file Supplementary_file_5.docx]

# Reanalysis Results After Excluding Studies at High Risk of Bias

**1 Reanalysis Results for PASI 75**

1.1 At 4 weeks of follow-up

1.2 At 8 weeks of follow-up

1.3 At 12 weeks of follow-up

1.4 At 16 weeks of follow-up

**2 Reanalysis Results for PASI 90**

2.1 At 4 weeks of follow-up

2.2 At 8 weeks of follow-up

2.3 At 12 weeks of follow-up

2.4 At 16 weeks of follow-up

**3 Reanalysis Results for Achieving Cleared or Almost Cleared Skin**

3.1 At 4 weeks of follow-up

3.2 At 8 weeks of follow-up

3.3 At 12 weeks of follow-up

**4 Reanalysis Results for DLQI**

**5 Reanalysis Results for Adverse Events**

5.1 AE

5.1.1 At 12 weeks of follow-up

5.1.2 At 16 weeks of follow-up

5.2 SAE

5.2.1 At 12 weeks of follow-up

5.2.2 At 16 weeks of follow-up

5.3 Discontinuation at 12 weeks of follow-up

5.4 Headache at 12 weeks of follow-up

5.5 Nasopharyngitis at 12 weeks of follow-up

5.6 Tractinfection at 12 weeks of follow-up
