# Supplementary material for: Efficacy and safety of interleukin inhibitors in the treatment of moderate-to-severe plaque psoriasis: a systematic review and network meta-analysis
Source: Front Med (Lausanne). 2026 Jul 23;13:1766348. doi: 10.3389/fmed.2026.1766348 (PMC13445711; doi:10.3389/fmed.2026.1766348)
Supplement: Supplementary file 5 [file Supplementary_file_6.docx]

**Supplementary Figure S1. Funnel plots of meta-analysis results for PASI 75. Subgroup analyses based on follow-up duration were performed. (A) At 4 weeks of follow-up; (B) At 8 weeks of follow-up; (C) At 12 weeks of follow-up; (D) At 16 weeks of follow-up.**

**Supplementary Figure S2. Funnel plots of meta-analysis results for PASI 90. Subgroup analyses based on follow-up duration were performed. (A) At 4 weeks of follow-up; (B) At 8 weeks of follow-up; (C) At 12 weeks of follow-up; (D) At 16 weeks of follow-up.**

**Supplementary Figure S3. Funnel plots of meta-analysis results for achieving cleared or almost cleared skin. Subgroup analyses based on follow-up duration were performed. (A) At 4 weeks of follow-up; (B) At 8 weeks of follow-up; (C) At 12 weeks of follow-up.**

**Supplementary Figure S4. Funnel plots of meta-analysis results for DLQI.**

**Supplementary Figure S5. Funnel plots of meta-analysis results for adverse events. Subgroup analyses based on different types of adverse reactions and follow-up duration were performed. (A) AE at 12 weeks of follow-up; (B) AE at 16 weeks of follow-up; (C) SAE at 12 weeks of follow-up; (D) SAE at 16 weeks of follow-up; (E) Discontinuation at 12 weeks of follow-up; (F) Headache at 12 weeks of follow-up; (G) Nasopharyngitis at 12 weeks of follow-up; (H) Tract infection at 12 weeks of follow-up.**
